# Operationalizing the Mind–Body Connection: Interoception via the Autonomic Nervous System

**DOI:** 10.3390/neurosci7010025

**Published:** 2026-02-12

**Authors:** Brittany Nackley, Bruce H. Friedman

**Affiliations:** Department of Psychology, Virginia Tech, Blacksburg, VA 24061, USA; bhfriedm@vt.edu

**Keywords:** interoception, autonomic nervous system, ANSception, integrative, multimodal, time series analysis

## Abstract

Traditional interoception research investigates cardioception, respiroception, or gastroception as a proxy for the sense of the body as a whole. These single-organ tasks sacrifice construct and ecological validity for a content validity that has been elusive. We propose that interoception is better captured by one’s sense of their own autonomic nervous system, or *ANSception*. The ANS integrates multimodal signals via lesser-myelinated neurons, making it an integral part of the interoceptive nervous system. Thirty-four participants moved a slider to reflect their perceived sympathetic activation (ANSception) while their physiology was monitored. Most participants reported integrating information from two or more organ systems during ANSception. The relationship between ANSception and physiology showed unique but often robust responses by condition and physiological measure. For example, one participant had a negative-to-positive-to-negative pattern for ANSception-EDA correlations from baseline to stimulus to recovery (r = −0.677; 0.657; −0.507, *p* < 0.001). Another participant had a strong positive correlation between their ANSception and blood pressure (r = 0.601, *p* < 0.001) during a five-minute reportedly meditative state. We propose that the role of interoception is to scan, integrate and manage information across organ systems, and we conclude that ANSception better captures this role than traditional single-organ tasks.

## 1. Introduction

Interoception, the sense of the internal state of the body, has been the subject of an explosion of research in the last decade. Interoceptive accuracy has been associated with positive mental health outcomes [1,2,3,4,5], while interoceptive deficits have been linked to psychopathology [6,7,8,9]. The unique neural structure supporting interoception was recently theorized to be the source of human consciousness [10], characterized as the “feeling mind”. Most literature recognizes the tremendous potential of interoception research yet simultaneously acknowledges the paucity of new perspectives [11,12]. Disparate findings across interoception measures have added confusion, leading one group to conclude that “[t]here is no such thing as interoception” [13].

Scholars agree that progress is stymied without a model that incorporates multiple modes of interoception [14,15,16,17,18,19]. In this paper, we introduce a new multimodal, integrative measure of interoception based on the autonomic nervous system (ANS) that operationalizes Singer and Damasio’s “feeling mind” of consciousness [10]. We then present empirical results using time series analysis to track moment-by-moment autonomic interoceptive sensations (ANSception) alongside four physiological measures.

### 1.1. Traditional Interoception Measures

The most common empirical measures of interoception are actually measures of cardioception, the ability to detect signals from the heart, usually indexing the timing of heartbeats. The heartbeat counting task (HCT [20,21]) requires participants to silently count their heartbeats during varying durations of under one minute. The heartbeat discrimination task (HDT; [22]) requires participants to determine whether the rhythm of an external stimulus matches their own internal heart rhythm.

Most research that challenges these foundational tasks has critiqued their content validity. HCT critics note systematic underestimation errors [5,23] and the confounding influence of educated guessing [24]. HDT critics have noted ideographic differences in cardiac cycle sensitivities [24] and the sheer difficulty of task success, with only one in three participants achieving the threshold to be a “heart rate detector” [25]. A meta-analysis found that those good at HCT had minimal overlap with those good at HDT [26].

In response to the shortcomings of traditional cardioception tasks, a wave of seemingly new and improved tasks has been proposed, primarily aimed at improving their content validity. Desmedt et al. [11] systematically reviewed 13 such measures introduced since 2015 and found that all measures could still be categorized as either “heart rate estimation paradigms” (HCT) or “synchronicity judgment paradigms” (HDT). The new HCT measures had the familiar problems with guessing contamination, while the new HDT measures had the familiar problems with task difficulty.

The authors did not identify the more detrimental problem with all cardioception measures: their poor construct validity and ecological validity. Decades of critiques of content validity alone have inadvertently distracted research focus from these more intractable problems. Construct validity concerns stem from the rung that cardioception inhabits on the pyramid of biological levels of organization. To operationalize interoception, the sense of the internal state of the body as a whole, researchers must focus more broadly than just on the heart, a single organ within a single organ system, among many organs and organ systems that all work together to yield the internal state of the entire organism. Regarding ecological validity, HCT and HDT tasks lack any known translational or evolutionary advantages [27].

There are two other traditionally researched modes of interoception beyond cardioception, but both suffer from poor content, construct, and ecological validity. Respiroception measures the ability to detect breathing variations; researchers typically occlude [28] or create resistance to [29,30] the breathing cycle, and then measure how well participants can detect these respiratory interruptions. Their content validity problems (exteroceptive cues and variations in breathing behavior; [11]) are overshadowed by issues with construct and ecological validity, namely their single system focus and the dubious portrayal of exogenous pulmonary interferences as valid inputs to interoception.

A third mode studied as interoception is gastroception, the sense of one’s gastrointestinal system. Researchers create gastric distensions or contractions in the esophagus [31], stomach [32] or colon [33] and participants are queried about the timing or intensity of their sensations. As with respiroception, exogenous perturbations bring into question the construct and ecological validity of gastroception research.

### 1.2. Neuroscience of Traditional Methods

The neuroscience of interoception sheds more light on the shortcomings of traditional methods. The insula is understood to be the ultimate neural center for interoception [34,35,36,37,38], fed by subcortical integration stations including the nucleus tractus solitarius (NTS), parabrachial nucleus (PBN), and the arcuate nucleus [10,39,40].

The tasks needed to complete traditional “interoception” research protocols generate additional activation in the somatosensory cortex during cardioception [41,42], respiroception [43,44], and gastroception [45,46]. By directing attention to only one organ, system, or body part, researchers have unwittingly diverted neural traffic away from the integrative interoceptive neural hubs to the somatosensory cortex with its somatotopic map.

Traditional interoception tasks are also prone to repetition suppression effects whereby neural receptivity is dampened with rhythmic, predictable stimuli such as heartbeats and breathing. Further, visceral receptors are widely distributed across organs but sparsely distributed within them [47]. Such neural mapping suggests that the most important function of interoception may be toggling awareness among the many organ systems to whichever system requires attention in the moment. Viewed from this perspective, traditional interoception research feels a bit like the blind men and the elephant. One team is focused on heart rate, another on respiration, when the larger picture requires interoception to sweep awareness across all systems and identify needed allostatic adjustments. Within-system precision misses this point.

Traditional interoception tasks follow what Singer and Damasio [10] recently described as “digital-like signaling”; these tasks favor rapid, specific, neural signaling at the expense of slower but more integrative processing. By contrast, the authors view the interoceptive nervous system as using “analogue-like processing”, which provides a real-time holistic view of one’s overall internal state, albeit at a slower pace. They point to several unique features of interoceptive neurons that make them integrative by nature: their minimal or absent myelination (allowing non-synaptic communication via ephaptic coupling, for example in the vagus nerve) and their close connection with circumventricular organs (CVOs) that can bypass the blood–brain barrier with critical messages such as homeostatic deviations.

With this backdrop, it becomes clearer how traditional interoception research tasks have been unintentionally dis-integrating at the neural level; they isolate sensations from an individual body part (heart, lungs, stomach), bypass the less myelinated/more integrated interoceptive hubs, and instead direct neural traffic to a dot on the homunculus. To compensate for this disintegration, researchers are now developing an artificial computational model to re-integrate in the computer lab what they disintegrated in the research lab. Hierarchical Bayesian models (HBMs [17]) are the latest example of this effort.

### 1.3. Putting the Body Back Together

**Figure 1:**
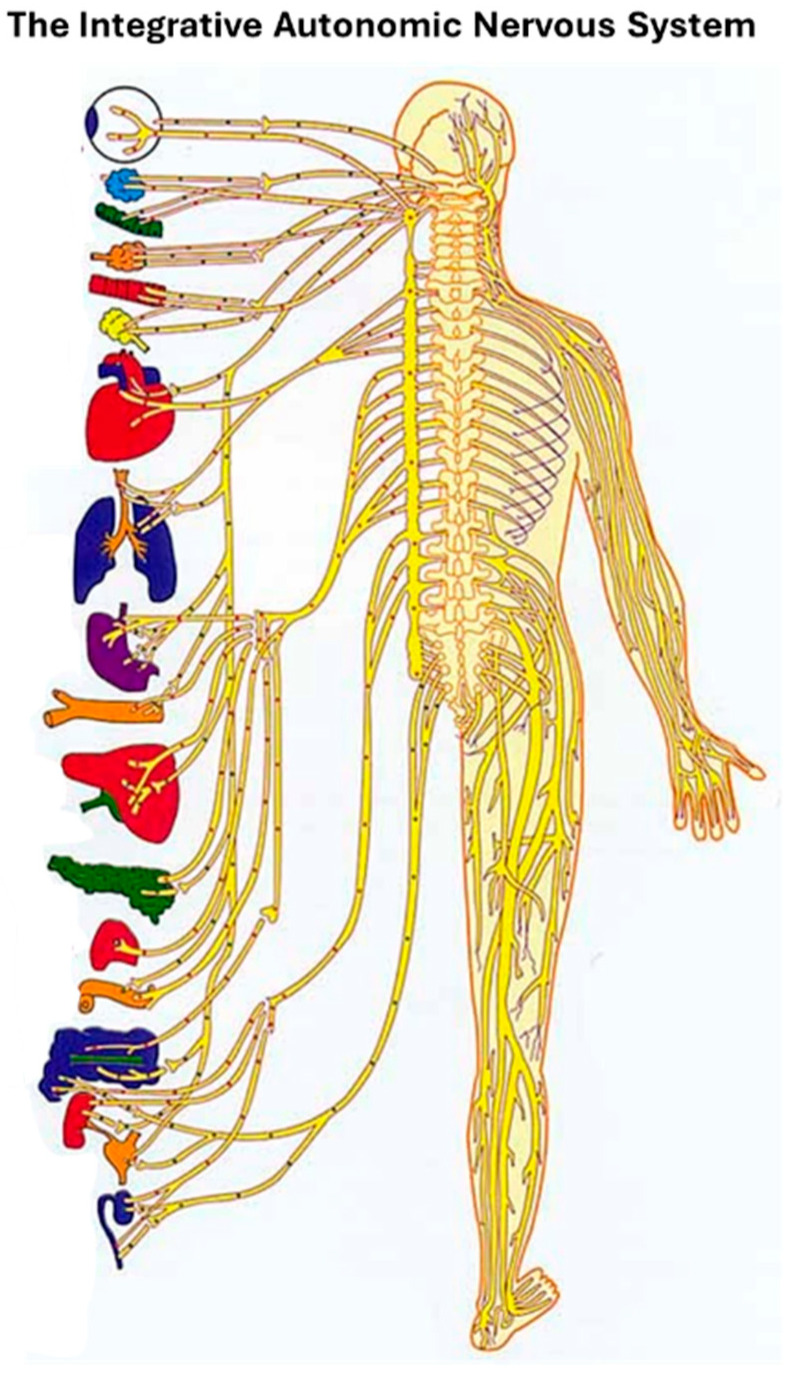
The ANS puts the interoceptive body back together. *[Image of autonomic nervous system], (n.d.)*.

Here we offer a new operationalization of interoception that indexes a person’s direct sense of their autonomic nervous system (ANSception). The ANS connects to multiple organs and systems, and it is part of the interoceptive nervous system [10,14]. The ANS is thus inherently integrative; it aggregates signals from disparate systems, including cardiovascular, respiratory, gastrointestinal, and more, as illustrated in Figure 1.

**Figure 2:**
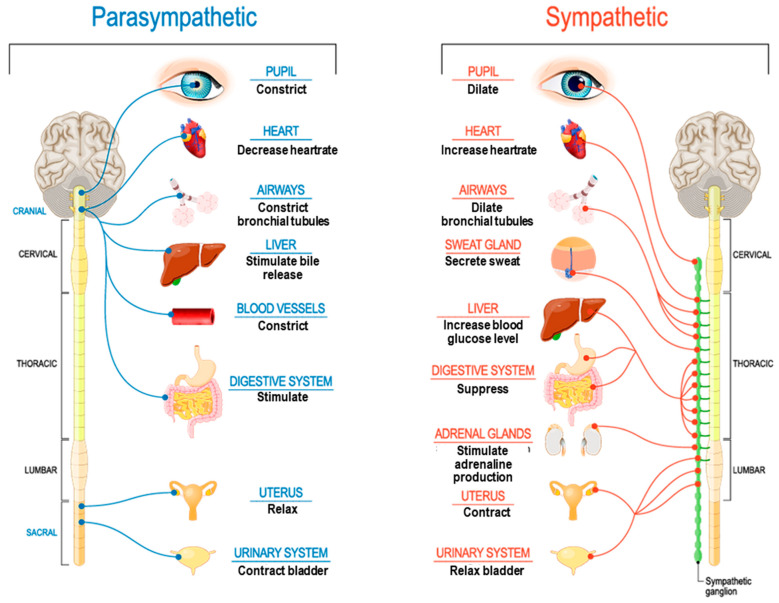
The ANS reconciles different systems moving in different directions. *[Image of autonomic nervous system], (n.d.)*.

Beyond its multimodal and integrative abilities, the ANS also reconciles signals that appear to move in different directions from different modes. For example, a strong increase in cardiovascular activity may be paired with a decrease in gastrointestinal activity. In isolation, these opposing forces may be confusing, but from the lens of the ANS, this pairing is natural. Sympathetic activation has the well-known effect of boosting cardiovascular and respiratory effort while suppressing gastrointestinal effort (see Figure 2).

Perhaps the most promising aspect of ANSception is that people already do it, consciously or subconsciously, and it feels natural. The ANS nets out all the disparate neural traffic to a single signal moving in a single direction. “I’m so wound up!” or “I’m just chilling.” Everyday language easily conveys one’s interoceptive reality more intuitively than an HBM.

This study introduces ANSception and offers an empirical proof of concept to demonstrate its accessibility in the laboratory. In the study, participants moved a slider to continuously reflect their perceived level of ANS activation while watching videos of varying intensity. Their physiology was simultaneously tracked in time series using the following four measures: cardiac pre-ejection period (PEP), electrodermal activity (EDA), heart rate (HR), and blood pressure (BP). We hypothesized that Pearson’s correlations between ANSception and each physiological measure, averaged over all participants, would be at least moderate (≥0.3) if not high (≥0.5). This research also replicated the HCT [21] as a comparative traditional measure of interoceptive accuracy. We hypothesized that performance on the HCT would be uncorrelated with ANSception.

## 2. Materials and Methods

### 2.1. Participants

The Virginia Tech Institutional Review Board approved this study, and all participants gave informed consent to participate. Virginia Tech undergraduate psychology students were recruited through fliers and a department online recruitment system. Recruits were screened for the following inclusion criteria: over 18 years, no high blood pressure or history of heart conditions, no neurological disorders or history of seizures, no medications that can affect the cardiovascular system and no use of tobacco products that exceeded 5 days per week.

In total, 219 participants completed the online survey, 135 met inclusion criteria, and 43 completed the study. Data from 9 participants were excluded due to missing blood pressure data, non-responsive EDA, or ECG artifact. Data from 34 participants (11 male) were ultimately included in the analysis. Mean age was 19.3 years (range: 18–22 years). Sixty-five percent were White, 18% Asian, 3% African American, 9% Hispanic/Latino, and 6% multi-racial.

### 2.2. Apparatus

**Figure A1:**
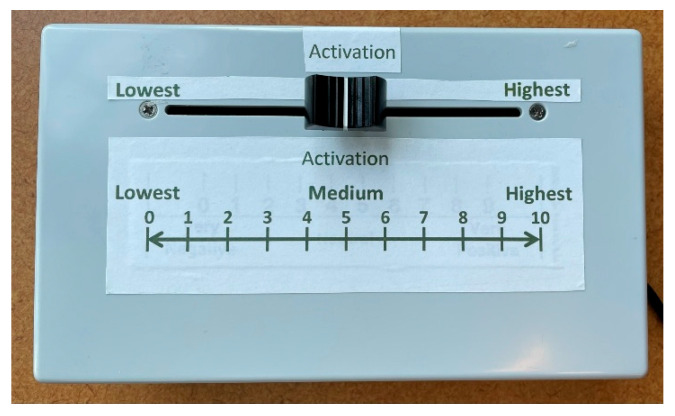
ANSception “Activation” slider.

**Figure A2:**
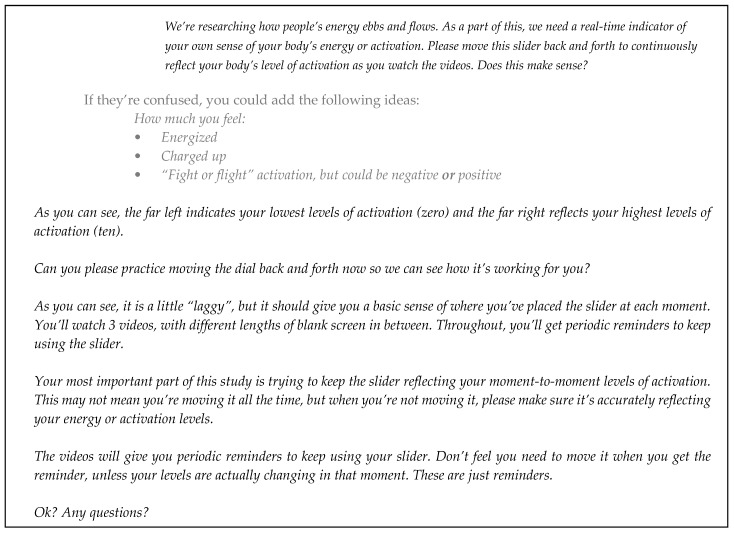
ANSception slider instructions.

ANSception was captured in real-time via the Biopac TSD115 Variable Assessment Transducer as the slider control (Biopac Systems, Inc., Goleta, CA, USA). This analog signal was transduced to a digital signal at 10 samples per second via the Biopac HLT100C High Level Transducer Interface module. This signal was then sent to the Biopac MP150 recording system. The slider was labeled “Activation”, with 0 to 10 and “Lowest” to “Highest” indicators. This allowed participants to report their moment-to-moment sense of their ANS activation throughout the study. Appendix A provides a photograph of the slider (Figure A1) and the script used to instruct participants on its use (Figure A2).

Physiological measures were gathered using the Biopac MP150 Data Acquisition System. An ECG Lead II signal was acquired at 2000 samples per second using the Biopac BioNomadix RSPEC. This used a three-electrode system, except the ground lead was eliminated because one was already used for the EDA, as noted below. One ECG electrode and lead pair was connected to the subject’s right collarbone, and the second pair was connected at the lowest left rib bone. Each electrode/lead pair was placed approximately equal distance from the heart.

Impedance cardiography (ICG) was collected at 2000 samples per second using the Biopac BioNomadix BN-NICO. Eight electrode/lead pairs were attached using four special-purpose paired electrodes with a configuration of two output leads on the right and left side of the neck, two input leads below the output leads, two input leads on the right and left side of the torso, and two output leads directly below the input leads on the torso.

Non-invasive continuous blood pressure (BP) was collected in a beat-to-beat fashion, and the signal was amplified using the CNAP^®^ Monitor 500 and Biopac NIBP100D system. A BP arm cuff was attached to the subject’s non-dominant arm and BP finger cuff attached to the proximal phalanx of the index and middle finger. The forearm was then placed on a small pillow in front of the participant for heart-level measurement of the signal.

EDA was obtained using the Biopac BioNomadix BN-PPGED. A pair of EDA electrodes was placed on the thenar and hypothenar eminences of the palm of the subject’s non-dominant hand.

### 2.3. Procedure

Interested participants completed an online pre-screen along with the battery of questionnaires for a separate portion of this study not reported here. Participants who passed the pre-screen were invited to enroll in the laboratory study. For the study, participants confirmed that they had abstained for 12 h from alcohol, 6 h from caffeine and other non-prescription drugs, and 2 h from eating and exercise [48]. They were then connected to the physiological equipment and completed another battery of questionnaires for a separate study. Participants were then instructed on the ANSception Activation slider to indicate their activation from moment to moment. (Figure A2 in Appendix A shows the script instructing participants on the use of the slider).

A series of video clips were then played as the main study (video details in Appendix B). A 5 min pre-stimulus video showed marine life accompanied by soothing music as a “vanilla baseline” condition [49]. Next, a 5 min video was shown as the main stimulus. This was an action scene from the movie *The Bourne Ultimatum* [50]. Next, a blank screen was shown for 90 s to allow ANSception without video stimulus. Finally, another 5 min marine life video was shown as post-stimulus. Participants were informed of the conclusion of the videos, but asked to continue tracking their activation for one more minute.

**Figure 3:**
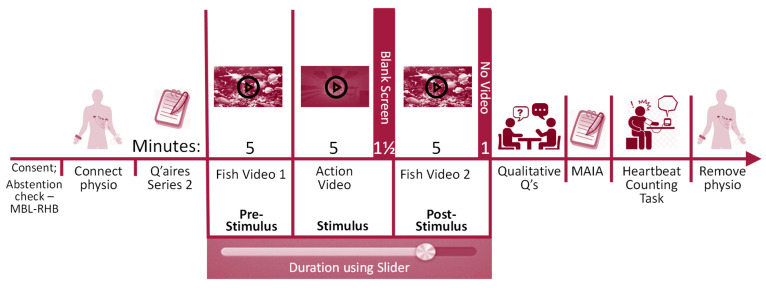
Study procedure.

Next, a series of qualitative questions were asked about participants’ experience with the videos and with tracking their body activation. The BP equipment was then removed and participants completed a questionnaire. They then completed the HCT followed by qualitative questions not reported on here. The remaining physiological equipment was removed and the study concluded. Figure 3 illustrates the procedure.

### 2.4. Data Reduction and Analysis

The Biopac AcqKnowledge Version 5.0 ECG Analysis software was used to classify the ECG, for subsequent calculation of HR and PEP. The ICG’s dZ/dt channel was classified using Biopac AcqKnowledge Version 5.0 software’s ICG Analysis with a specification to apply the R-to-C polynomial method outlined in Lozano et al. [51]. Using AcqKnowledge, PEP was calculated as the time between the QRS-onset in the ECG channel to the B-point in the dZ/dt channel, which follows methodology from Sherwood et al. [52]. PEP was then visually inspected, and outlier values were removed via linear interpolation with the nearest accurate values.

For the traditional HCT, the modified accuracy index [53] was calculated from reported heartbeats for each trial as follows:

1 − (|*n*beats_real_ − *n*beats_reported_|)/((*n*beats_real_ + *n*beats_reported_)/2)

Each participant’s average accuracy score was then calculated by averaging the accuracy index over all six trials, as used by Garfinkel et al. [53], based on Hart et al. [54]. The raw difference between reported and actual heartbeats was also calculated for each trial for each participant and then averaged over all six trials for a net estimate error.

ANSception as interoceptive accuracy was operationalized as the Pearson correlation coefficient between ANSception and each of the physiological measures.

Time series analysis began by calculating a single mean value for each cardiac cycle for all measures, which down-sampled without data loss. The physiological data were further smoothed by applying centered, moving averages as detailed in Nackley et al. [55]. Moving averages are critical for time series analysis of high lability physiological data, revealing the signal from within the noise. Using this technique, we averaged across 35 cardiac cycles for PEP, HR, and BP, and 8 cycles for EDA. Supplementary Figure S1 in Supplement SA illustrates this methodology with PEP.

## 3. Results

Correlation analysis was first conducted on an aggregate basis, averaging correlations across all participants and all conditions, to determine if any one physiological measure was consistently correlated with ANSception. No single measure was significant across all participants. We then examined each physiological measure for each participant individually. There were many significant correlations at this level, although which measure was significant differed by participant. Finally, informed by these correlation analyses, we conducted extensive time series analyses combined with qualitative interviews, which uncovered a wealth of information about how individuals experience and report their sense of their autonomic nervous system.

### 3.1. Aggregate Correlation Analysis

Correlation analysis began by taking the Pearson correlation coefficient between EDA and ANSception for each participant. This produced one correlation number for each participant, for a total of 34 correlation coefficients for the 34 participants. Then we took the mean of these 34 correlation numbers to obtain a single average correlation coefficient to reflect the overall relationship between EDA and ANSception. We then repeated this process for the other three physiological measures. There was no significant relationship at this aggregated level; results are shown in Table 1.

### 3.2. Heartbeat Counting Task

The modified accuracy index for the traditional HCT was below the 50% chance level that would be expected from uninformed guessing (M = 0.47, SD = 0.31). On average, participants severely underestimated their total number of heartbeats (M = 17.7, SD = 10.4). The HCT modified accuracy index was also compared with the ANSception accuracy index in Table 2.

### 3.3. Correlation Analysis by Participant

**Figure 4:**
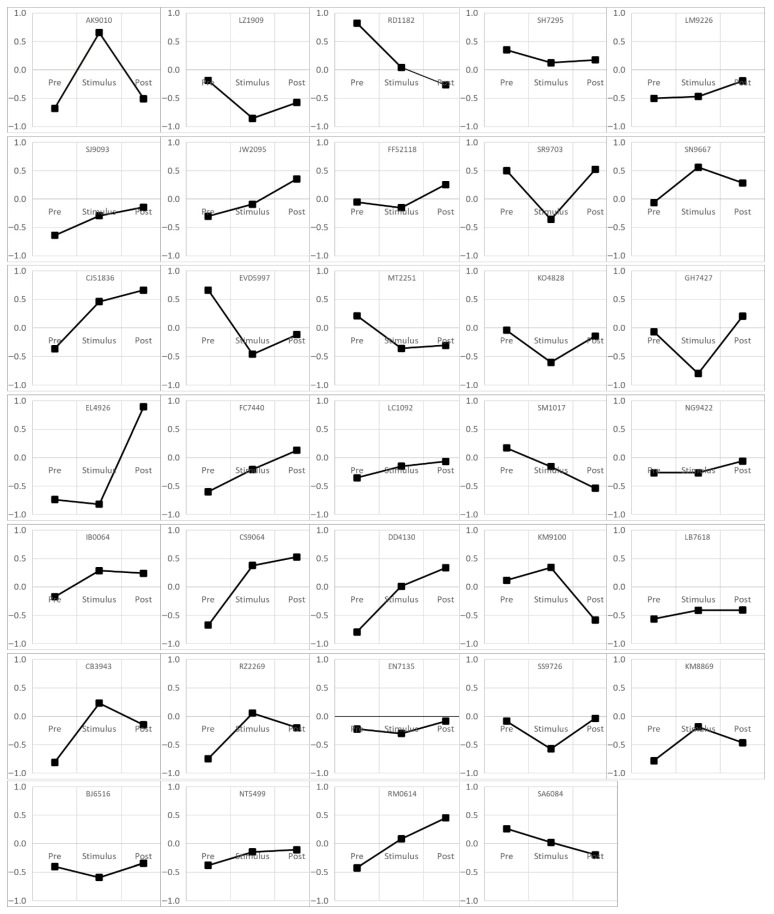
ANSception-EDA correlations by subject and condition (*p* < 0.001 for all).

Pearson’s correlations were calculated between ANSception and each physiological measure for each participant and condition. Figure 4 shows these correlations between ANSception and EDA.

Statistically significant, moderate to strong correlations were observed for many participants and conditions, although the directionality of these correlations varied widely. Many participants had at least one 5 min condition with moderate correlations (88% with absolute value > 0.3) or even strong correlations (68% with absolute value > 0.5). Similar ideographic variations were found for the remaining physiological measures. Correlations between BP and ANSception were similarly strong to the EDA-ANSception relationship. Most participants (85%) had at least one condition with moderate correlations (absolute value > 0.3) and nearly half of the participants (47%) had at least one condition with strong correlations (absolute value > 0.5). The correlations for PEP-ANSception and for HR-ANSception were weaker than those for EDA-ANSception. Correlations between ANSception and PEP, HR, and BP are shown in Supplement SB in Figure S2, Figure S3, and Figure S4, respectively.

### 3.4. Qualitative Feedback

After the video and slider experience, participants were asked qualitative questions about their experience: 76% of the participants noticed their heart beating; further, 91% noticed their breathing during the experience. Many elaborated on how this affected their interoceptive processes. All participants who noticed their heartbeat also noticed their breathing, marking 76% of participants who used both cardiovascular and respiratory inputs into their interoceptive assessments. Some participants noticed additional psychosomatic feedback such as lump in the throat (3 participants), butterflies in the stomach (4 participants), tightness in the jaw (6 participants) and dry mouth (8 participants).

Before participants were specifically prompted with the questions listed above, they were asked a broader question about any specific strategies they used to track their body activation. Table 3 shows all answers to this open-ended question.

Regardless of strategy, all participants successfully completed the ANSception task and did not have any confusion or difficulty with the task instructions.

### 3.5. Time Series Analysis

**Figure 5:**
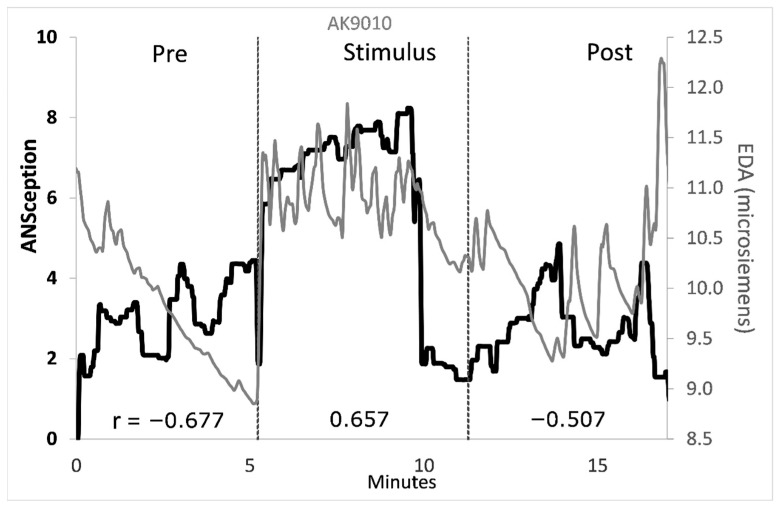
Time series of ANSception and electrodermal activity for a selected participant. ANSception (black) with electrodermal activity (gray). Correlation coefficients shown at bottom for each condition.

Time series graphs were created for each participant portraying ANSception with each ANS measure. The degree of correspondence between each pair of measures represents a more complete operational definition of interoceptive accuracy. Graphs with all pairs of measures for all participants are shown in Supplement SC. Informed by the prior correlation analysis, selected graphs were chosen to examine the time series detail behind large correlation fluctuations, especially those with direction changes across conditions. Figure 5 illustrates the ANSception-EDA relationship for a participant who had large changes in correlations.

**Figure 6:**
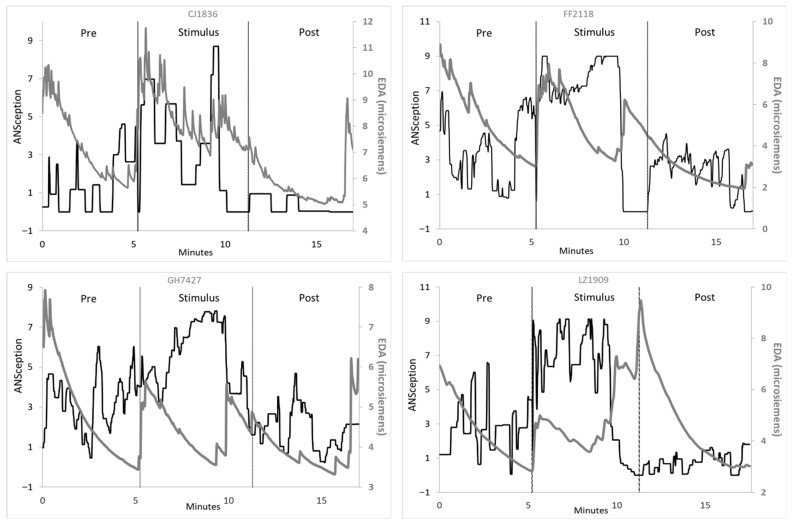
EDA shows novelty–habituation pattern for each condition. ANSception (black) with electrodermal activity (gray).

A novelty–habituation pattern emerged with EDA, where it spiked rapidly at the onset of each new condition, and then slowly dropped over the course of the condition as the novelty wore off and the participant appeared to habituate to the condition. This pattern is illustrated in Figure 6, which shows four representative participants.

Many participants including the four above had an additional novelty spike around the 10 min mark. This represented the moment the action video stopped and a blank screen was shown on the stimulus computer for 90 s. While the researchers considered this to be a continuation of the “Stimulus” period, the participants appeared to experience this change as a new condition, with novelty spikes at the 10 min point.

Some participants also showed a fifth novelty spike one minute before the end of the experiment. This was when the researcher announced the end of the videos and asked each participant to continue tracking their body activation for one more minute. There was an absence of new computer stimuli for this duration, but participants’ EDA responded as if it was a new experience.

**Figure 7:**
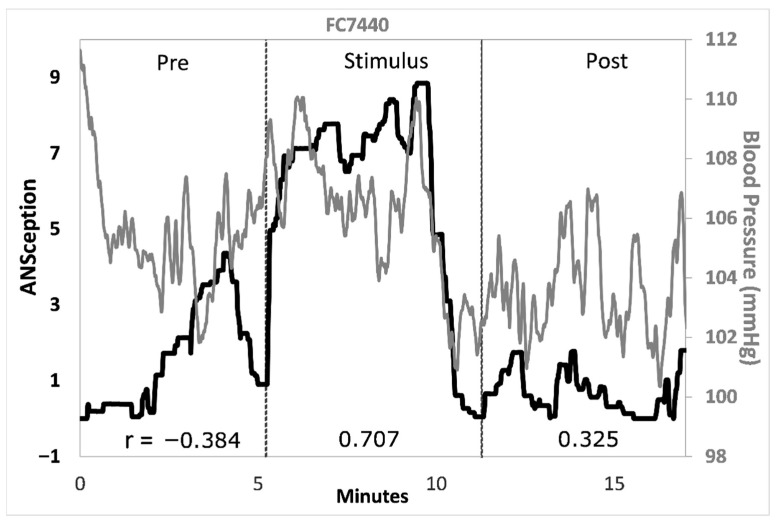
Time series of ANSception and blood pressure for a selected participant. ANSception (black) with blood pressure (gray). Correlation coefficients shown at bottom for each condition.

Blood pressure was another physiological measure that had strong correlations with ANSception for many participants. Figure 7 shows this relationship for a selected participant.

### 3.6. Mixed Method Analysis

**Figure 8:**
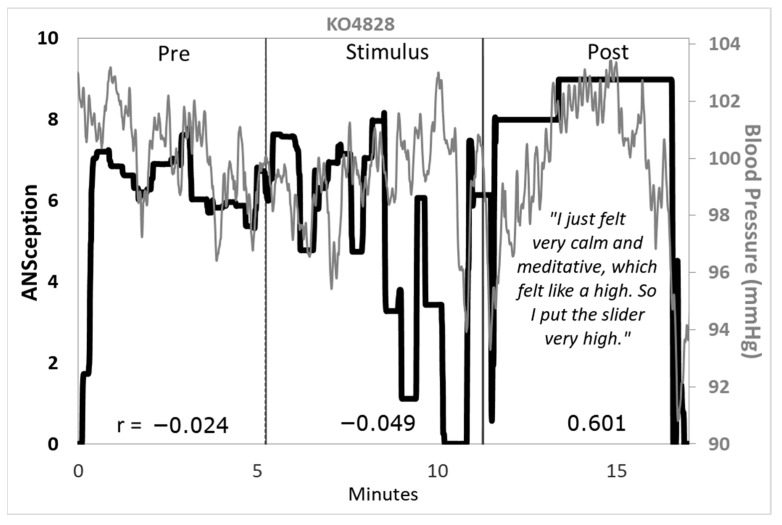
ANSception and blood pressure for a selected participant. ANSception (black) with blood pressure (gray). Correlation coefficients shown at bottom for each condition.

Combining qualitative information from Table 3 above with quantitative time series analysis provided further insights. The most striking example involved a participant who specifically addressed their experience during the post-stimulus period. The time series analysis revealed a statistically significant, strong positive correlation between this participant’s ANSception and blood pressure, shown in Figure 8.

## 4. Discussion

Contrary to our first hypothesis, correlations between ANSception and each physiological measure were below moderate levels when averaged over all participants. In line with our second hypothesis, modified HCT accuracy was mostly uncorrelated with ANSception accuracy, except ANSception-PEP, which had a statistically significant moderately positive relationship.

At the participant level, statistically significant moderate and strong correlations were common between ANSception and each physiological measure albeit different ones for different participants. Time series analysis revealed the moment-to-moment changes behind each correlation data point.

Qualitative analysis revealed that over three-quarters of participants used more than one organ system for ANSception, noticing both heartbeats and breathing. Additional somatic indicators included sweaty palms, body tension, dry mouth, and “butterflies” in the stomach. Participants also used behavioral clues (widening eyes, tapping foot, twitching), emotional clues (“feeling intrigued”, gauging how “into it” they felt, noticing if a scene “took me off-guard”), and cognitive meta-awareness of the aforementioned clues as well as noticing distraction, or how much one was thinking or “overthinking it”. This evidence suggests that interoception as defined by ANSception is naturally multimodal, integrating mind and body into one sense.

Mixed method analysis identified an intriguing case study; one participant’s ANSception-BP correlation became strongly positive during a reportedly meditative condition that “felt like a high”. Time series analysis offered visibility into changes over time during this surprisingly long duration of mind–body alignment. Meditative states may yield higher interoceptive accuracy, and a peaceful mind–body alignment can feel like a high, revealing the paradoxical pairing of calm state and high activation.

EDA had a novelty–habituation response to each condition; even the absence of videos produced EDA novelty spikes at the onset of the absence, followed by a slow habituation drop during each condition. When stimulus novelty had a temporal sequence opposite of participants’ ANSception, there were predictably negative correlations between these measures. Interpreting EDA-ANSception relationships thus requires caution, accounting for stimulus novelty patterns.

HR and BP were originally included in this study to replicate studies using these measures as proxies for sympathetic activation, even though they are under dual SNS-PNS influence. Surprisingly, both measures showed stronger correlations with perceived “Body Activation” than PEP did, and they avoided EDA’s novelty–habituation confound.

Long-standing autonomic research provides a strong foundation upon which to develop an ANS-based definition of interoception. Research on homeostasis and allostasis describe the ANS response to detected or predicted deviations from physiological equilibrium [56,57,58,59,60]; ANSception models *how* one might predict and fulfill energy needs based on the interplay between exteroception of the external environment and interoceptive assessment of internal capacity and requirements. Relatedly, ANSception incorporates bi-directional top-down and bottom-up neural pathways [34] through the ANS’s role in energy management [58,59]. Our research illustrates how ANSception integrates affective approaches of emotional awareness, adding weight to prior research into the connection between emotion and interoception [47,61,62,63]. ANSception also explicitly modeled the interplay between conscious and nonconscious states [34] via participant slider movements; participants acknowledged gauging “how into it” they felt.

### 4.1. Limitations

This study included only college-aged participants, which may not generalize to a wider population. However, traditional heartbeat interoception research has not conclusively shown advantages to younger adults in interoceptive accuracy. Research would be needed to see how ANSception abilities vary over the lifespan.

This study did not address within-subject reliability; our research design only tested participants on a single laboratory visit. More research is needed to determine the reliability of the ANSception findings over time for participants.

This study did not isolate or examine the potential influence of the parasympathetic nervous system (PNS) nor did it examine respiration, measures which could provide more insight about ANSception. Participants were also instructed to remain seated and hold still during the study. This necessity for obtaining clean physiological signals is in direct opposition to the need for more movement which would lend further ecological validity. Any physiological arousal that participants experienced was not able to be “metabolized” in service of responding to experiences of threat or challenge. This contrived experience could suppress the natural reciprocal and iterative nature of interoception and action.

### 4.2. Future Directions

Building on the foundation just presented, researchers could examine neural activation while participants perform ANSception tasks. Related research tracking physiological responses may add a time series measure of PNS such as respiratory sinus arrhythmia to reveal more about the ANS response in interoception. Advanced methodology such as the Sympathetic Activation Index (SAI) and Parasympathetic Activation Index (PAI) [55,64] may provide further time series precision about the ANS response.

Studies allowing for more participant mobility may better model the afferent–efferent feedback cycle as in allostasis [58]. For example, a haunted house stimulus [65] could allow exteroceptive environmental feedback to interact with afferent interoceptive feedback to guide efferent responses. Virtual reality may be a more laboratory-friendly way to address this idea, using combat-style interactive gaming to simulate an environmental threat.

## 5. Conclusions

We introduced ANSception as a new operationalization of interoception that advances construct and ecological validity, and we provided preliminary empirical evidence supporting its use in the laboratory. Participants intuitively integrated information from two or more organ systems into this single measure. We thus propose a revised role for interoception; to scan, integrate and manage information *across* organ systems, a role for which it is uniquely equipped neurologically [10,47]. Fittingly, perhaps the first known use of the term “sympathy” in reference to what is now known as the ANS came from Galen (A.D. 130–200 [66]) to describe “the co-operation or co-ordination of organs”. Traditional interoception research testing single-organ precision requires the interoceptive nervous system to perform tasks for which it is neurologically poorly equipped.

Scholars have been calling for a new measure of interoception; ANSception answers that call and fulfills a long list of research requirements. ANSception explicitly describes a nervous system process, a requirement embedded in the updated definition of interoception [17]. This new measure also meets the need for moment-to-moment mapping, modeling interoceptive changes over time [17,19]. ANSception addresses the need to incorporate multiple modes, organs and systems, a requirement universally identified by interoception scholars [10,14,15,17,18,19,67]. ANSception further integrates disparate signals arising from disparate systems moving in disparate directions into a single ANS signal. ANSception also aggregates signals with different physiological timescales/frequencies/amplitudes (e.g., heart rate: 0.5–3.3 Hz, respiratory rate: 0.08–1 Hz, gastric contractility: 0.05–0.1 Hz) [10,17] into a single 0-to-10 scaled measure. Additionally, ANSception explains *how* the brain integrates across organ systems via existing literature tracing afferent and efferent ANS neural networks [10,34]. Contrasting with HBMs, ANSception presents a model built on human intuition about one’s own ANS. Finally, training on ANSception has the potential for translational benefits such as improved body and emotional literacy [68], whereas training on heartbeat detection has no demonstrated translational benefits [27].

We hope that this study provides the missing link for interoception research, and we look forward to investigations using ANSception to map a clearer picture of the mind–body connection.

## Figures and Tables

**Table 1:** Aggregated mean correlations.

ANSception Correlations with Physiological Measures			
	Mean	St. Dev.	*p*
**EDA**	0.03	0.31	0.87
**PEP**	0.1	0.26	0.58
**BP**	0.06	0.32	0.72
**HR**	0.01	0.23	0.96

**Table 2:** HCT accuracy index vs. ANSception accuracy index for each physiological measure.

Correlations: ANSception Accuracy vs. HCT Accuracy with Physiological Measures		
	Mean	*p*
**EDA**	0.07	0.69
**PEP**	0.36	0.04 *
**BP**	0.03	0.89
**HR**	0.15	0.4

**Note**. n = 34. * *p* < 0.05.

**Table 3:** Open-ended question with participant answers.

***Were there any specific physical and mental strategies you used to keep track of your body activation?***	
AK9010	EL4926
*See how I was breathing*	*I was feeling my finger pulse a lot and my breathing as well.*
LZ1909	LC1092
*I would tense up or feel my eyes were getting wider (pupils dilating), I felt more activation.*	*I could feel my breathing slightly different, I felt more at rest at some points.*
RD1182	FF52118
*Yawns would cause me to slide it down. If there was fast music or something funny, I would think “I’m into this”, so I would slide it up.*	*Not generally. I like to think that I can typically tell when my heart rate increases, so it was based off of that and my breathing.*
NG9422	IB0064
*I was keeping in check with my breathing, I was checking if my palms were sweaty, or if my hands were getting cold.*	*No, just considering when I was most stressed or excited. There was a lot of stress, excitement, fear.*
LM9226	SH7295
*I tried to think how into it I was and if it was making me tired or not.*	*Twitching, heartbeat in my chest, tightness in my chest.*
SJ9093	SM1017
*I tried thinking about it, but then I think that I started overthinking, so maybe it was higher because I thought about it. Without thinking, I might have been more accurate.*	*Focusing on breathing and if something took me off guard, especially with action film. When something took me off-guard, it caused a stronger reaction, as they were more captivating, or at a faster pace.*
JW2095	CS9064
*When I was breathing, the pushing of my chest against the band allowed me to feel my own heart rate. The rate I was breathing. My mental state at the time.*	*I just sort of focused on how I breathed at certain instances, or whether or not my heart was beating faster, and if it was the same as when I started.*
FC7440	DD4130
*I just made sure I was focusing on the screen and not looking at slider.*	*Keeping track of my heartbeat.*
SN9667	MT2251
*Just looking at my breathing and the thoughts I was having. Some of the videos made me think back to an incident that I had. So I felt it a little, but then I tried to take my mind off of it. The very first time saw the fish swimming around—reminded of snorkeling accident.*	*I moved the slider up when they first started fighting, but then moved it back down because it wasn’t catching me off guard anymore. I would move the slider up every time something new happened, but the move it back down when it got old.*
SR9703	LB7618
*If I felt a feeling within my body, I tried to express that with my hand, but it was hard to track and indicate at the same time.*	*Keeping track of times I tensed up or held my breath for a second or felt my heart going faster*
CJ51836	CB3943
*I tried staying mindful about my heart rate and my breathing.*	*No*
EVD5997	RZ2269
*Yes, once my thoughts started to stress me out, I realized my breathing was more short paced, so I took a deep breath and calmed myself down.*	*I was just monitoring my breath. When I was concentrating on my breathing more, that’s how I knew it was going up a little bit.*
KM9100	BJ6516
*Trying to maintain the same breathing rhythm so that I could track my heart rate. So I was using my heart rate and how much attention I was paying to certain parts of the video.*	*I focused on my breathing. And how focused I felt like I was on the video. Some parts I was more focused on than others. The action video I was more focused on.*
KM8869	SS9726
*The thing that said “keep using your slider”. It was easier on the action one because I was a little more awake compared to the fish one which was more calming and put me to sleep a little bit. The music and the sounds from the action video made me feel more awake compared to just the sounds of the water and fish.*	*During the fish videos, I was focusing on my breath because I was getting really sleepy. During the Jason Bourne video, I was trying to monitor how engaged I was with the scene, and the anticipatory factor.*
GH7427	KO4828
*No, I just kept doing it. I noticed that when I first saw the ocean video, my heart rose a lot and I felt really intrigued. Then when it went from the first to second video, it rose a lot. For the second fish video I was less activated. I was thinking of the activation, so I was more conscious of my body while I was watching the videos. I felt really tense with second (action) video. For the second ocean video, there was less activation than the first time—I almost felt bored because I had seen it before.*	*I gauged how calm I felt. I felt times of calmness during the fish scenes* vs. *the dark screen, which was worse than the action. I watch “Game of Thrones”, so any action I’m used to. During the fish scene, I just felt very calm and meditative during the second fish scene, which felt like a high, it was very peaceful, pleasant, happy, so I put the slider very high.*
SA6084	EN7135
*How calm I’m feeling.*	*No*
NT5499	RM0614
*If I noticed that I started to get a little nervous, I felt my heartbeat more, I knew my fight or flight was kicking in and I moved the slider*	*Not really—during the first fish video with the big ocean background —I’m kind of scared of oceans—I moved slider when my attention was grabbed.*

## Data Availability

The original contributions presented in this study are included in the article/Supplementary Materials. Further inquiries can be directed to the corresponding author.

## Supplementary Materials

The following supporting information can be downloaded at: https://www.mdpi.com/article/10.3390/neurosci7010025/s1, Supplement SA. Moving Average Methodology for PEP: Figure S1: Pre-Ejection Period (PEP) is smoothed to reveal underlying trends using 35 cycle moving average; Supplement SB. Correlation Graphs by Participant: Figure S2: PEP-ANSception Correlations by Subject (*p* < 0.001 for all); Figure S3: HR-ANSception Correlations by Subject (*p* < 0.001 for all); Figure S4: BP-ANSception Correlations by Subject (*p* < 0.001 for all). Supplement SC. Time Series Graphs by Participant: Figure S5: Time Series Graphs by Participant.

## Appendix B. Stimulus Videos

Pre-stimulus and post-stimulus videos depicted marine life with a slow tempo soundtrack; sample here: https://www.youtube.com/watch?v=O9AaqmMv4HUt.

The stimulus video was a 5 min clip from the action movie *The Bourne Ultimatum* (a portion of which can be seen here: https://www.youtube.com/watch?v=DUd5RPVDjPY).
