# Supplementary material for: Operationalizing the Mind–Body Connection: Interoception via the Autonomic Nervous System
Source: NeuroSci. 2026 Feb 12;7(1):25. doi: 10.3390/neurosci7010025 (PMC12922123; doi:10.3390/neurosci7010025)
Supplement: Supplementary file 1 [file neurosci-07-00025-s001.zip › neurosci-4097090-supplementary.pdf]

Supplement A

Moving Average Methodology for PEP

Figure S1: Pre-Ejection Period (PEP) is smoothed to reveal underlying trends using 35 cycle moving average

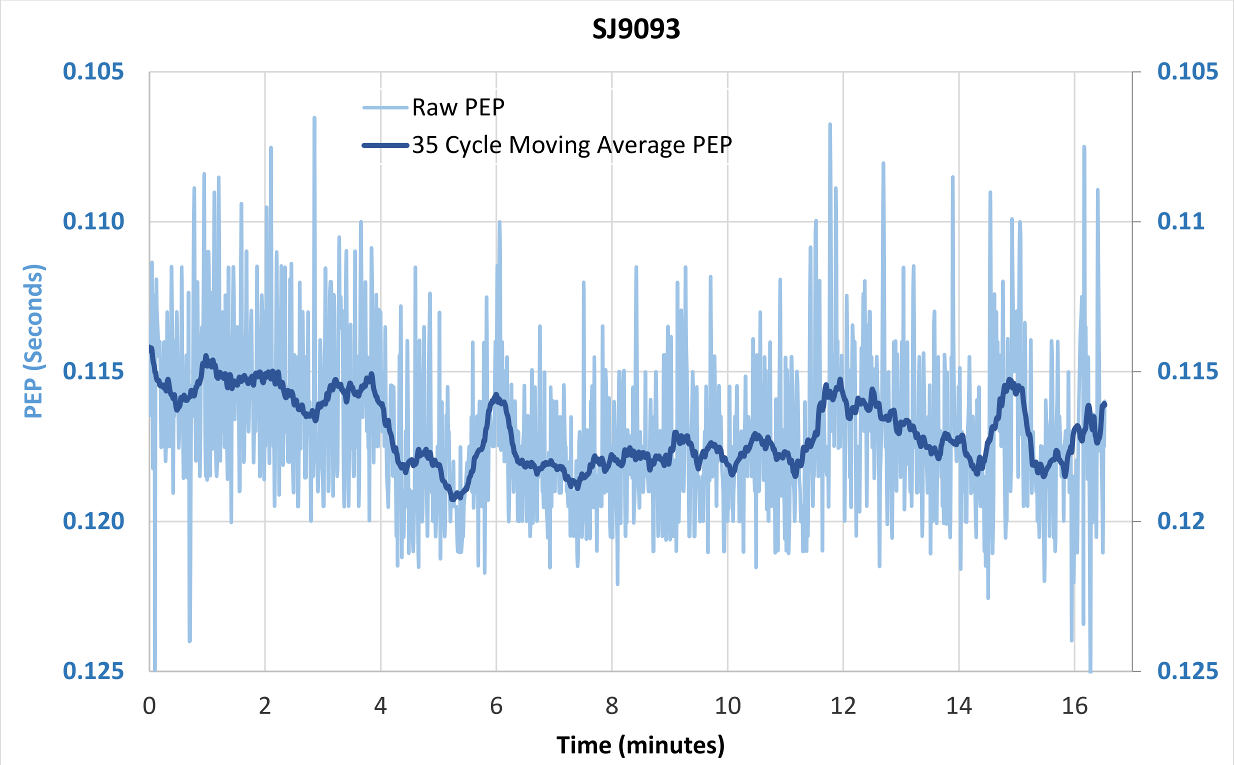

PEP is depicted on reverse scale to reflect directionality of underlying sympathetic activation

Supplement B - Correlation Graphs by Participant

Figure S2: PEP-ANSception Correlations by Subject (p < 0.001 for all)

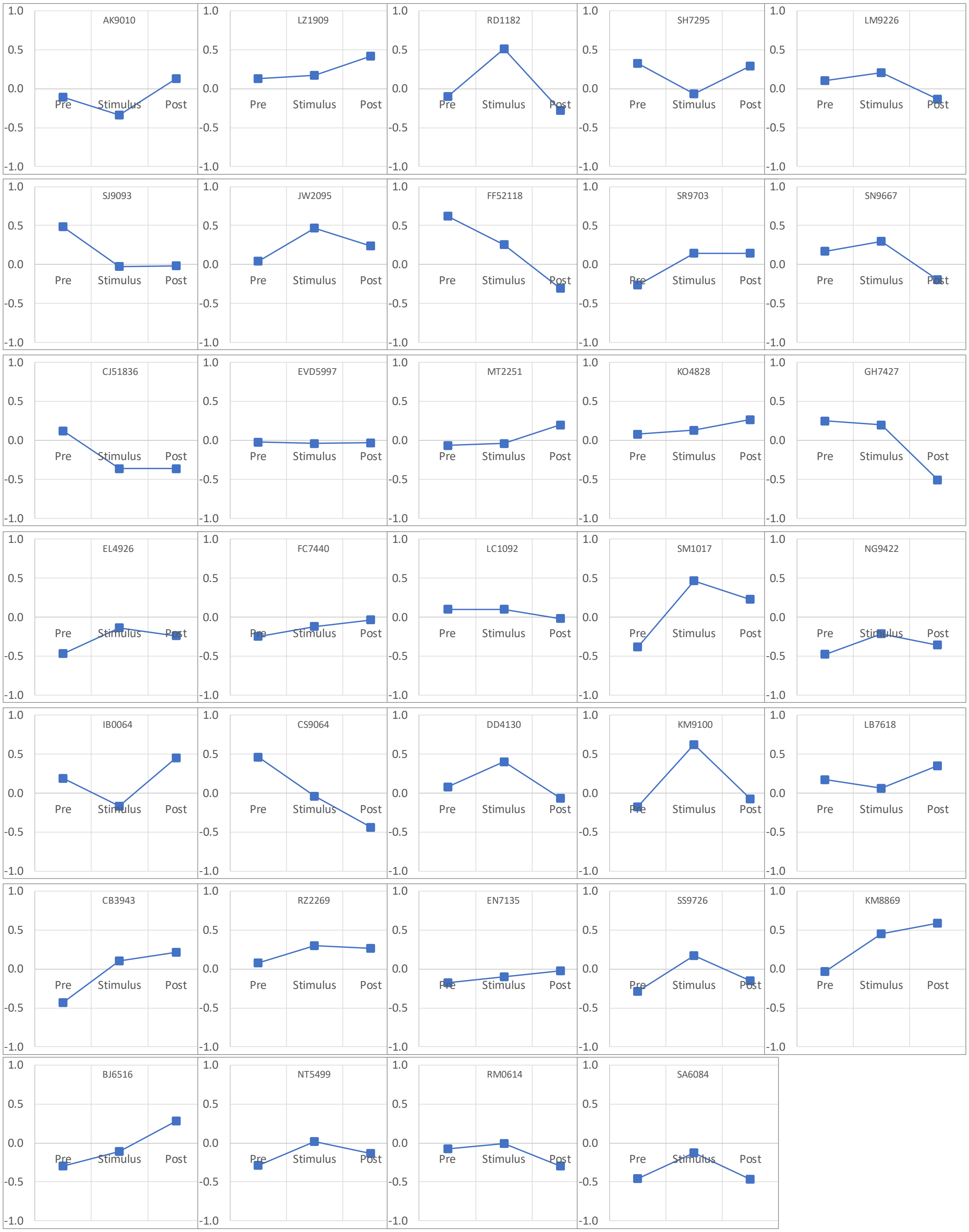

Figure S3: HR-ANSception Correlations by Subject ($p < 0.001$ *for all*)

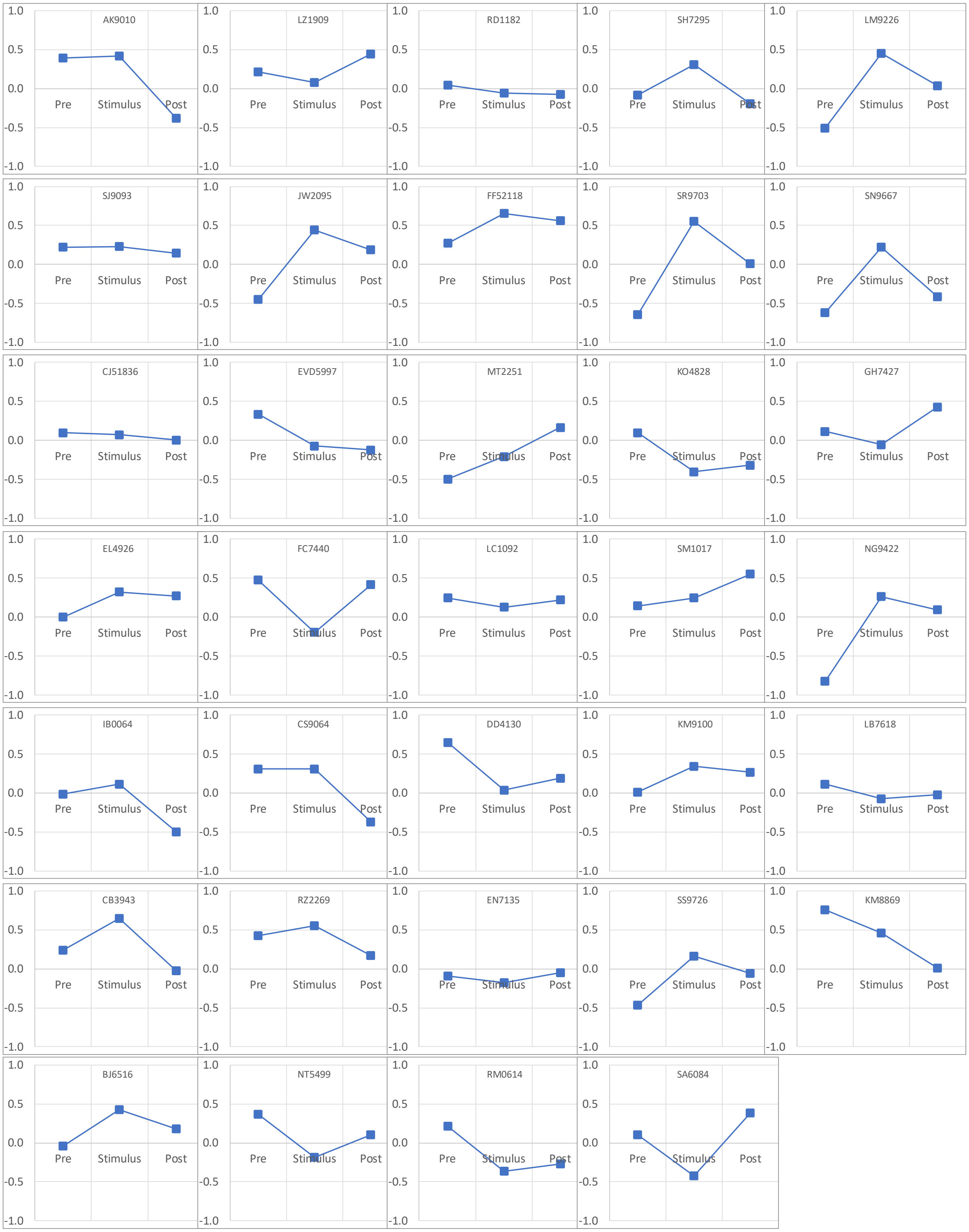

Figure S4: BP-ANSception Correlations by Subject (p < 0.001 for all)

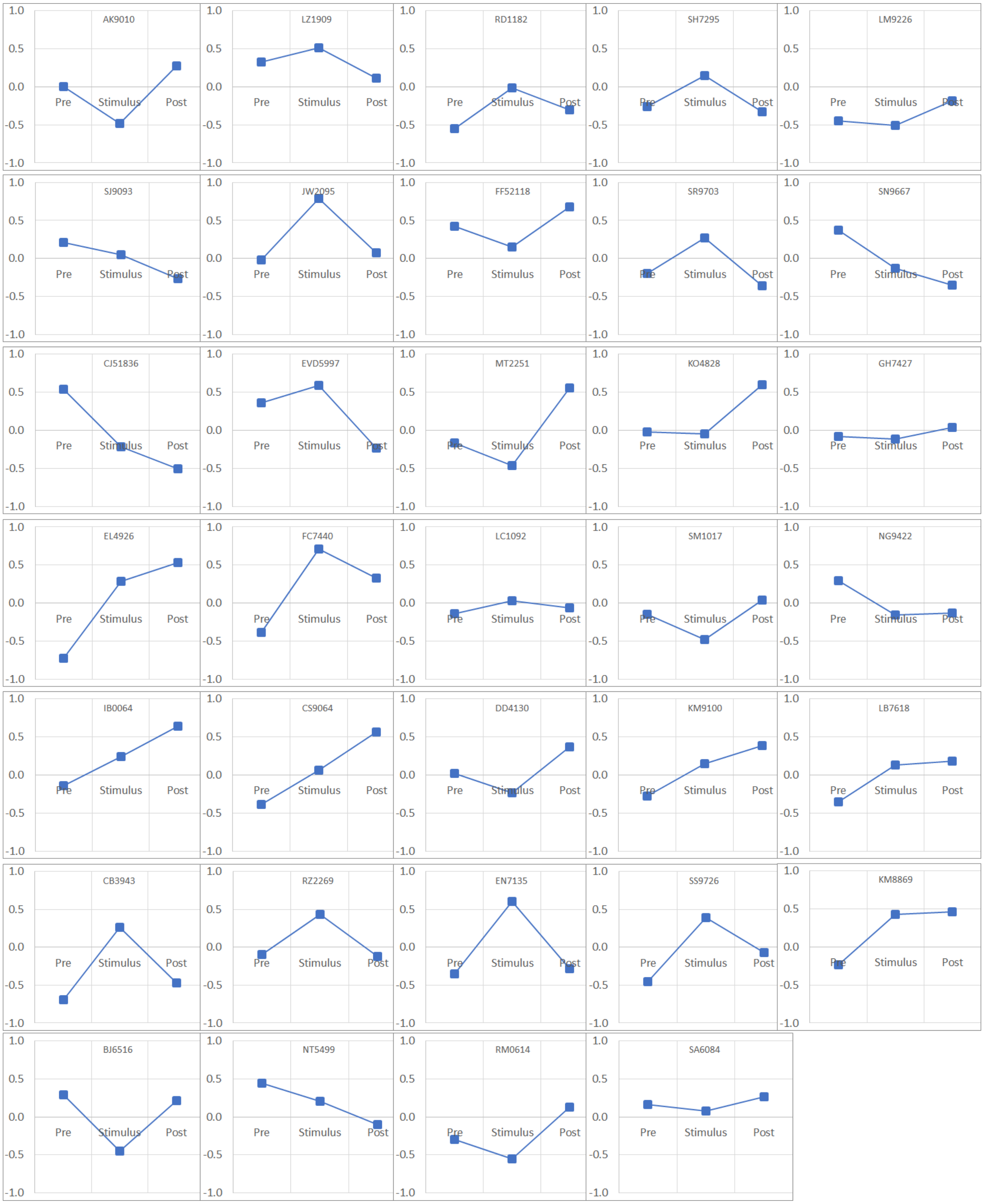

Supplement C – Time Series Graphs by Participant

Figure S5: Time Series Graphs by Participant

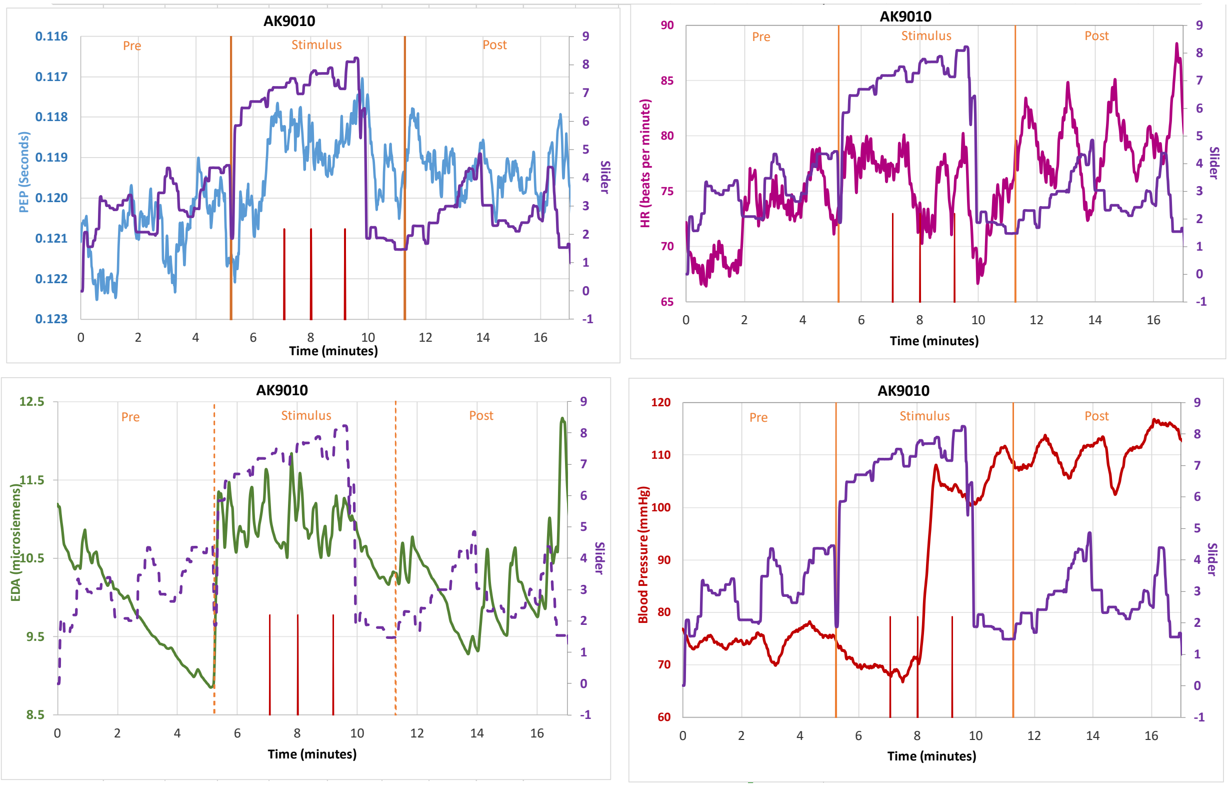

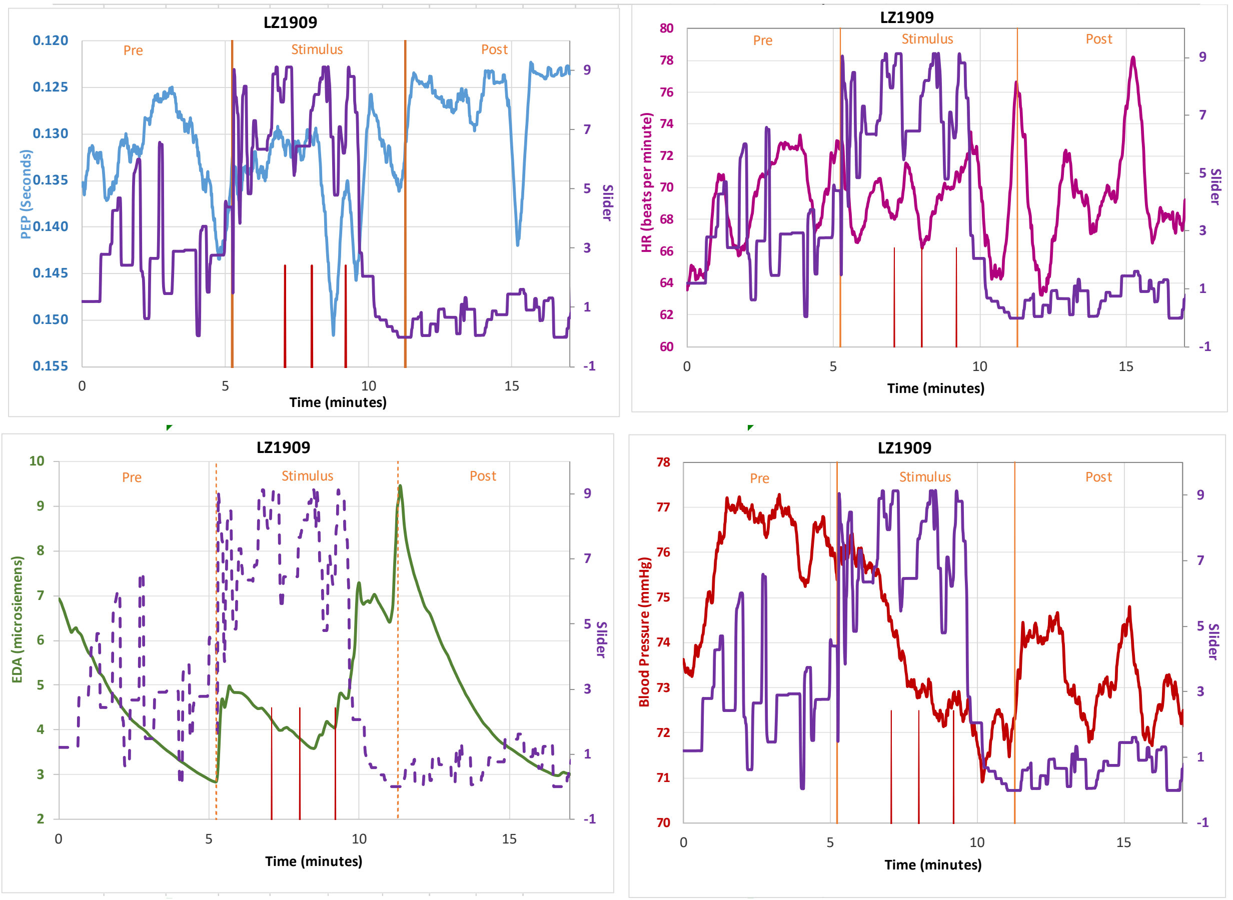
LZ1909
Pre
Stimulus
Post
PEP (Seconds)
0.120
0.125
0.130
0.135
0.140
0.145
0.150
0.155
Slider
9
7
5
3
1
-1
0
5
10
15
Time (minutes)
LZ1909
Pre
Stimulus
Post
HR (beats per minute)
80
78
76
74
72
70
68
66
64
62
60
Slider
Time (minutes)
LZ1909
Pre
Stimulus
Post
EDA (microsiemens)
10
9
8
7
6
5
4
3
2
Slider
Time (minutes)
LZ1909
Pre
Stimulus
Post
Blood Pressure (mmHg)
78
77
76
75
74
73
72
71
70
Slider
Time (minutes)

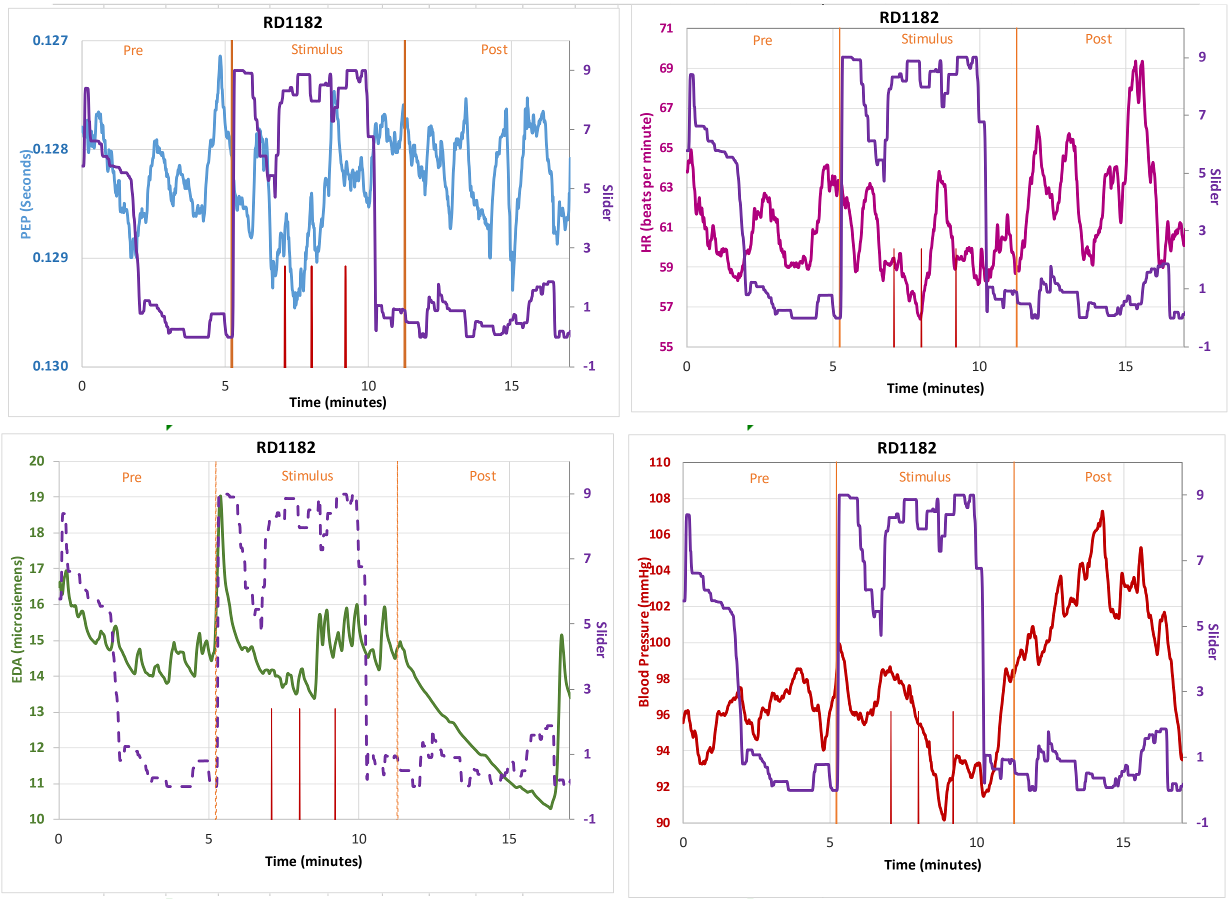
RD1182
Pre
Stimulus
Post
PEP (Seconds)
0.127
0.128
0.129
0.130
Slider
9
7
5
3
1
-1
Time (minutes)
0
5
10
15
RD1182
Pre
Stimulus
Post
HR (beats per minute)
71
69
67
65
63
61
59
57
55
Slider
Time (minutes)
RD1182
Pre
Stimulus
Post
EDA (microsiemens)
20
19
18
17
16
15
14
13
12
11
10
Slider
Time (minutes)
RD1182
Pre
Stimulus
Post
Blood Pressure (mmHg)
110
108
106
104
102
100
98
96
94
92
90
Slider
Time (minutes)

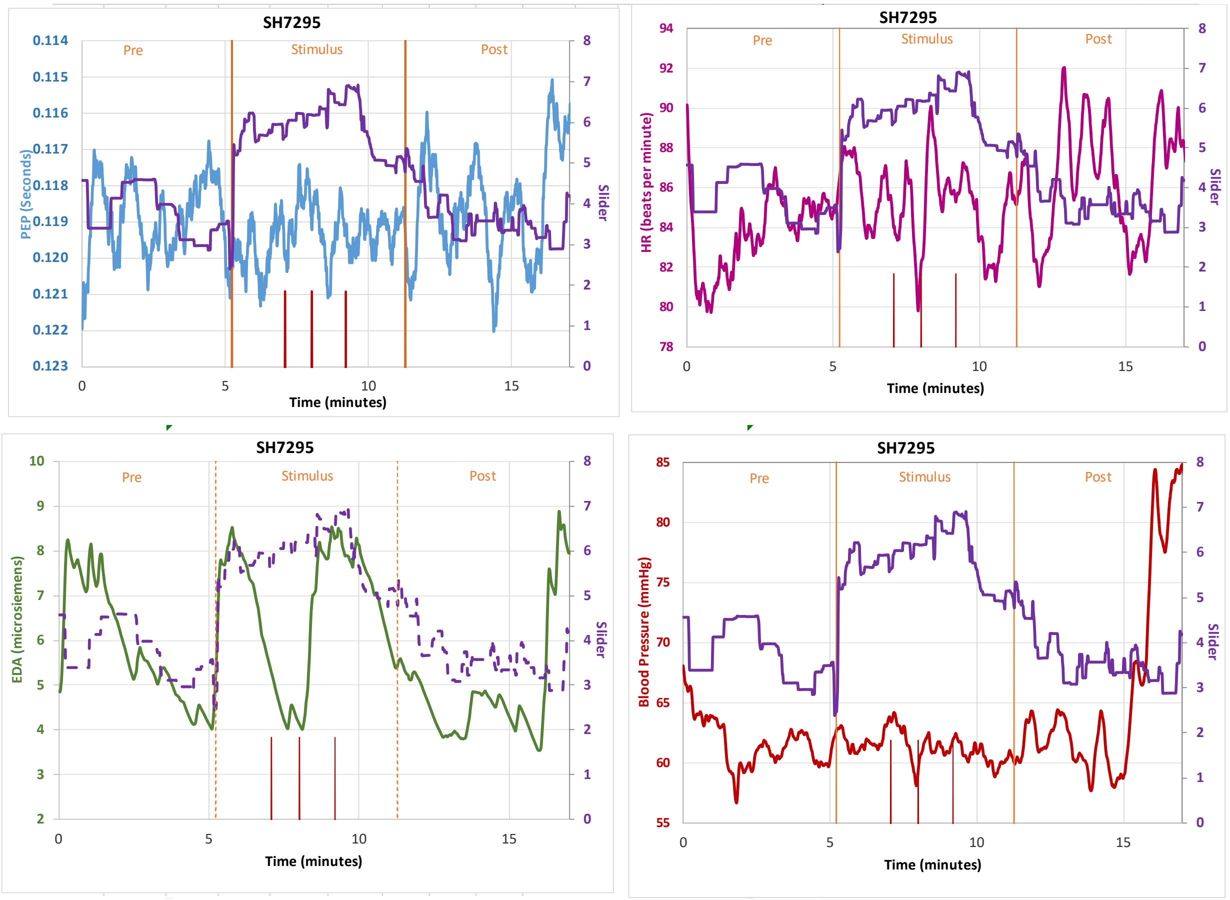
SH7295
Pre
Stimulus
Post
PEP (Seconds)
0.114
0.115
0.116
0.117
0.118
0.119
0.120
0.121
0.122
0.123
Slider
0
5
10
15
Time (minutes)
SH7295
Pre
Stimulus
Post
HR (beats per minute)
94
92
90
88
86
84
82
80
78
Slider
Time (minutes)
SH7295
Pre
Stimulus
Post
EDA (microsiemens)
Slider
Time (minutes)
SH7295
Pre
Stimulus
Post
Blood Pressure (mmHg)
85
80
75
70
65
60
55
Slider
Time (minutes)

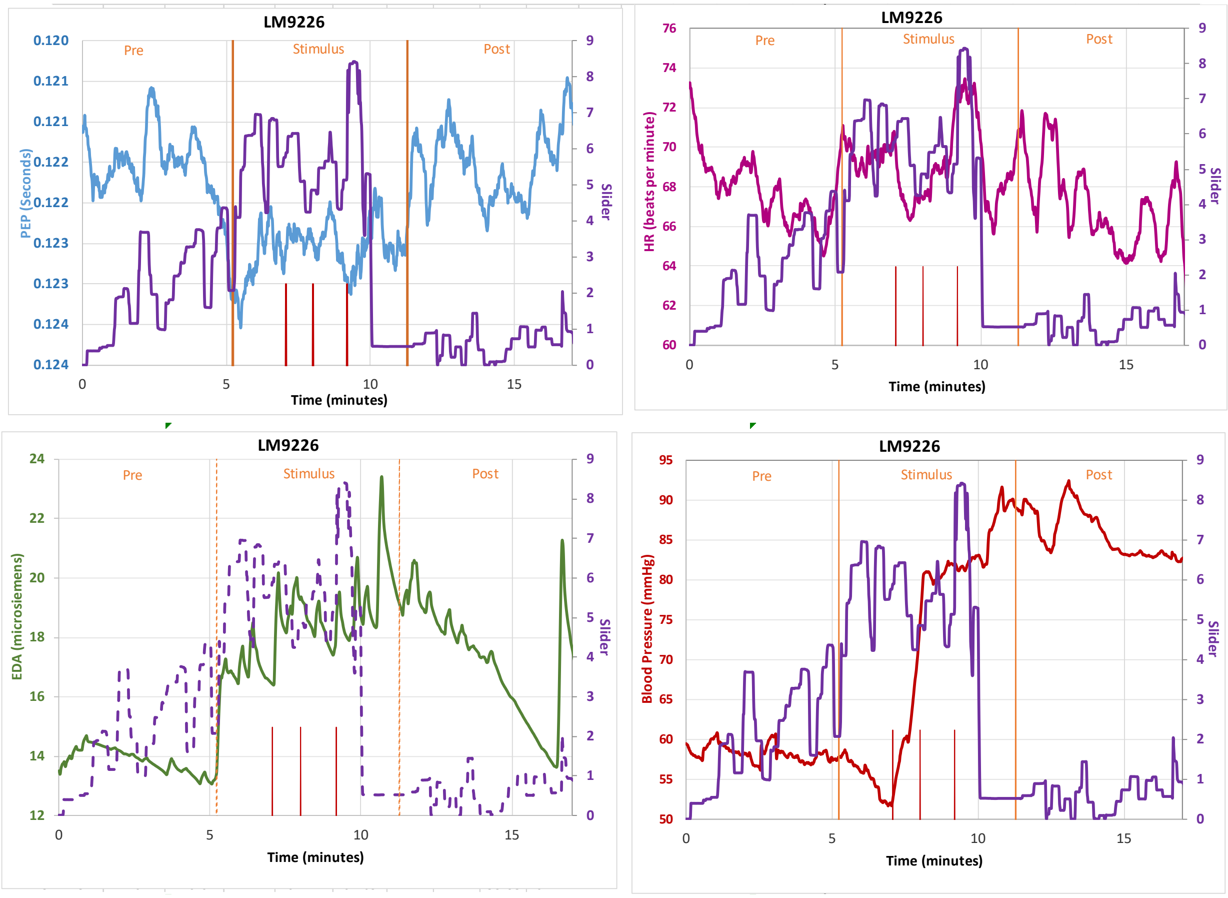
LM9226
Pre
Stimulus
Post
PEP (Seconds)
0.120
0.121
0.121
0.121
0.122
0.122
0.123
0.123
0.124
0.124
Slider
Time (minutes)
LM9226
Pre
Stimulus
Post
HR (beats per minute)
Slider
Time (minutes)
LM9226
Pre
Stimulus
Post
EDA (microsiemens)
Slider
Time (minutes)
LM9226
Pre
Stimulus
Post
Blood Pressure (mmHg)
Slider
Time (minutes)

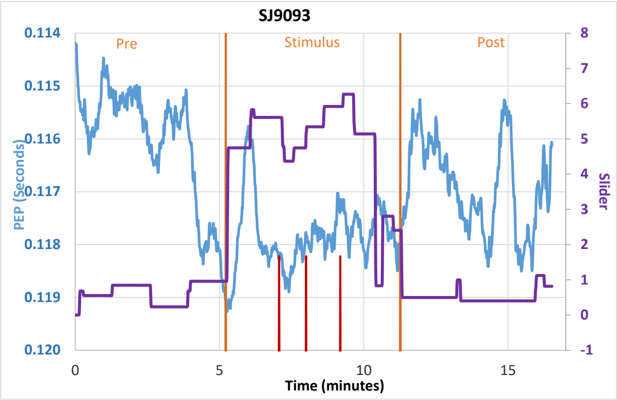
SJ9093
Pre
Stimulus
Post
PEP (Seconds)
Slider
Time (minutes)

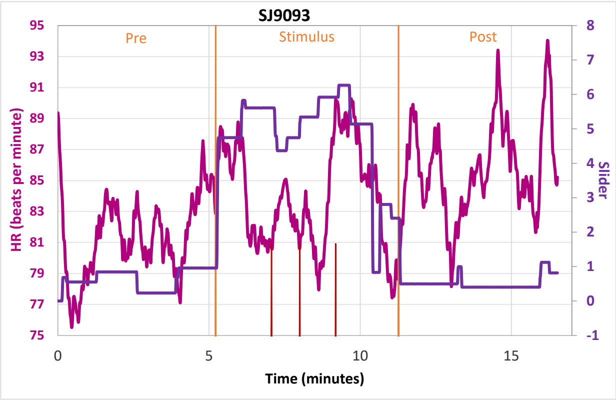
SJ9093
Pre
Stimulus
Post
HR (beats per minute)
Slider
Time (minutes)

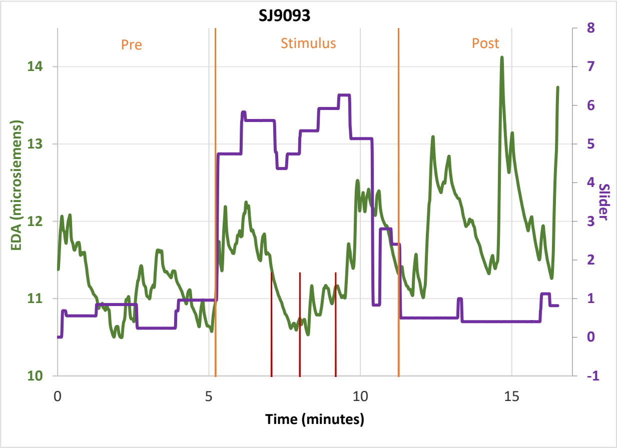
SJ9093
Pre
Stimulus
Post
EDA (microsiemens)
Slider
Time (minutes)

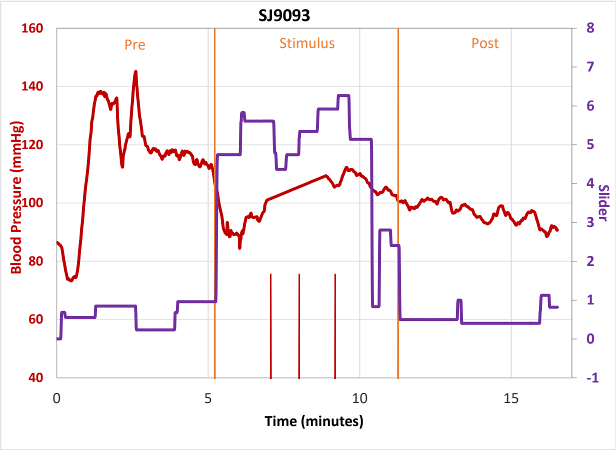
SJ9093
Pre
Stimulus
Post
Blood Pressure (mmHg)
Slider
Time (minutes)

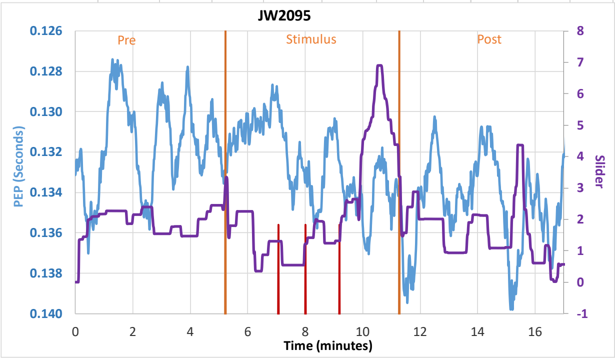
JW2095
Pre
Stimulus
Post
PEP (Seconds)
Slider
Time (minutes)

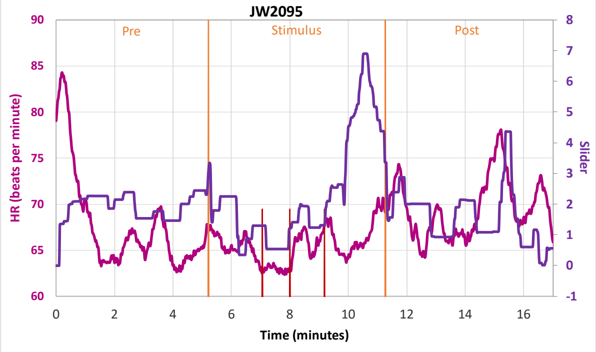
JW2095
Pre
Stimulus
Post
HR (beats per minute)
Slider
Time (minutes)

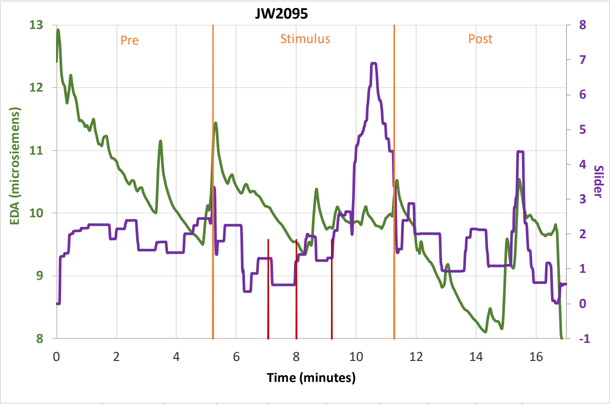
JW2095
Pre
Stimulus
Post
EDA (microsiemens)
Slider
Time (minutes)

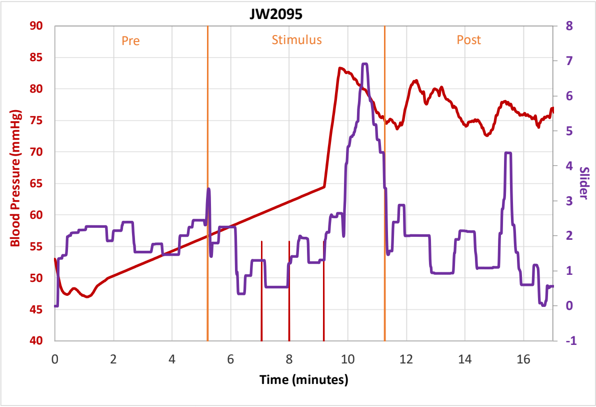
JW2095
Pre
Stimulus
Post
Blood Pressure (mmHg)
Slider
Time (minutes)

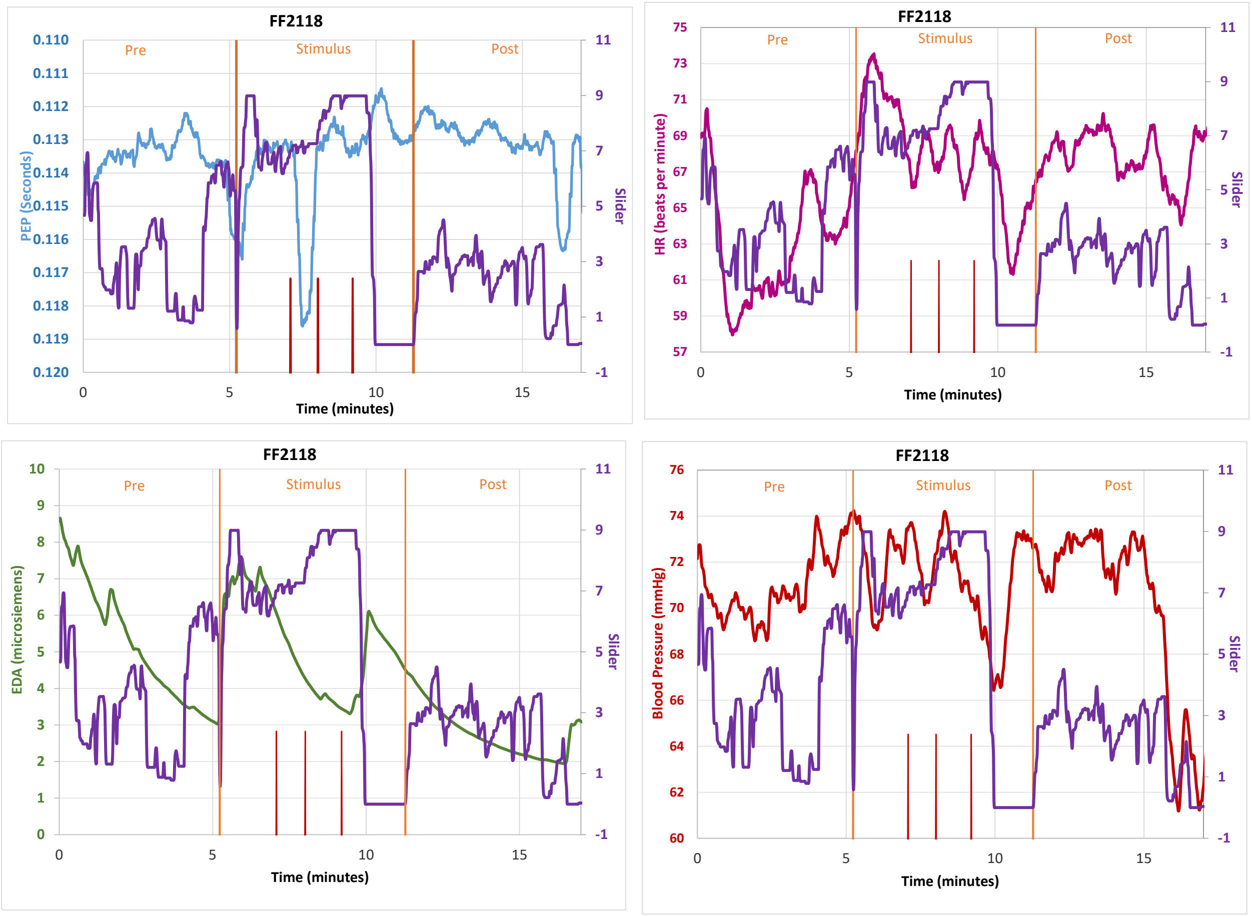
FF2118
Pre
Stimulus
Post
PEP (Seconds)
Slider
Time (minutes)
FF2118
HR (beats per minute)
Slider
Time (minutes)
FF2118
EDA (microsiemens)
Slider
Time (minutes)
FF2118
Blood Pressure (mmHg)
Slider
Time (minutes)

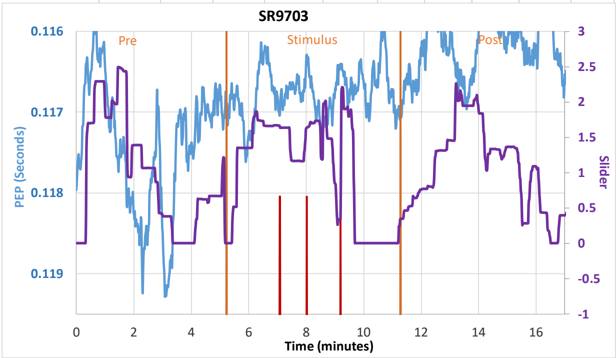
SR9703
PEP (Seconds)
Slider
Pre
Stimulus
Post
Time (minutes)

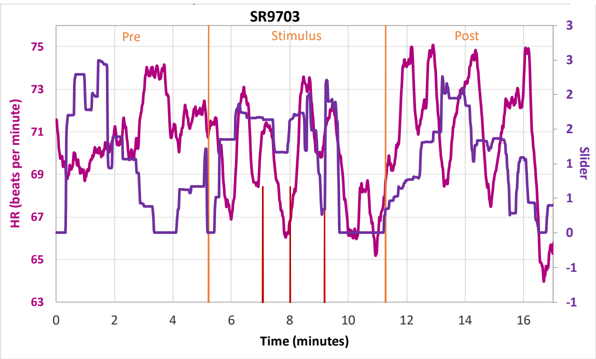
SR9703
HR (beats per minute)
Slider
Pre
Stimulus
Post
Time (minutes)

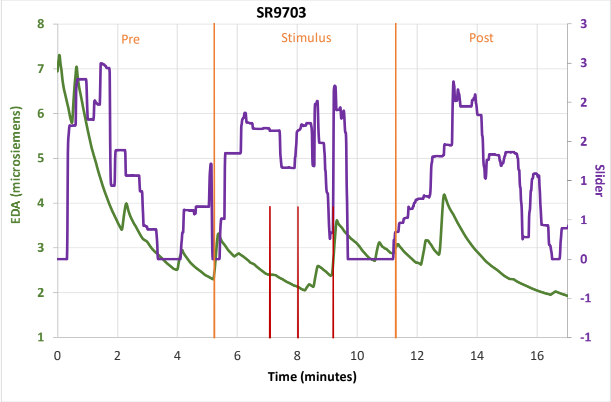
SR9703
EDA (microsiemens)
Slider
Pre
Stimulus
Post
Time (minutes)

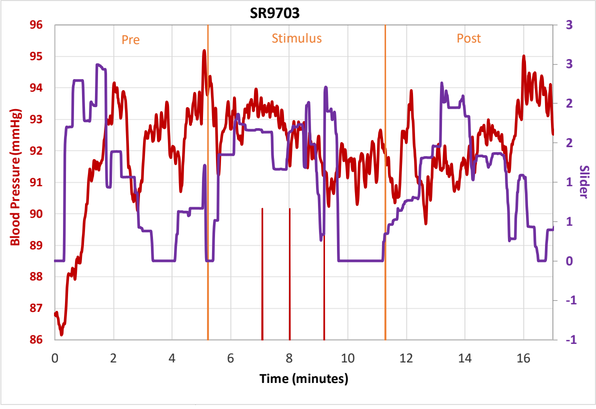
SR9703
Blood Pressure (mmHg)
Slider
Pre
Stimulus
Post
Time (minutes)

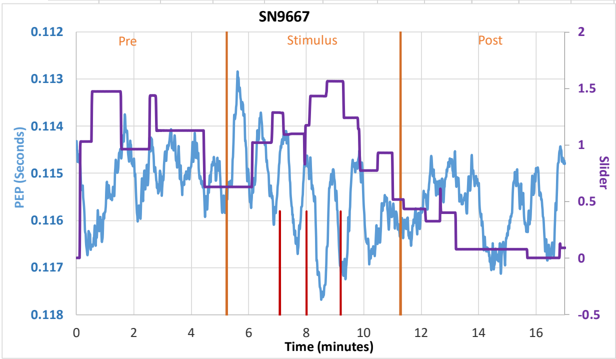
SN9667
PEP (Seconds)
Slider
Pre
Stimulus
Post
Time (minutes)

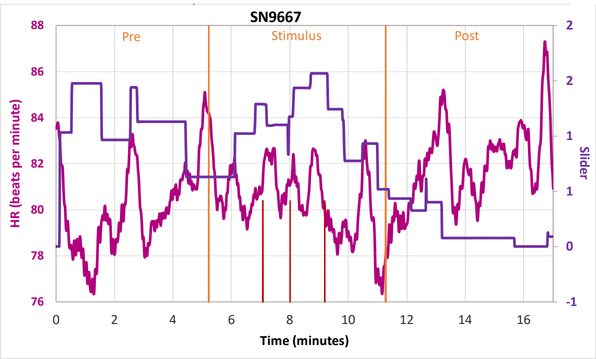
SN9667
HR (beats per minute)
Slider
Pre
Stimulus
Post
Time (minutes)

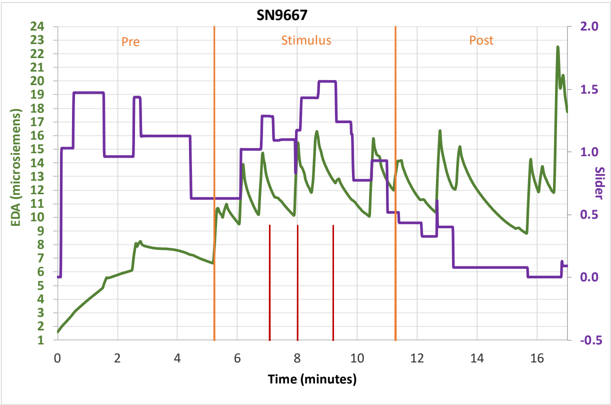
SN9667
EDA (microsiemens)
Slider
Pre
Stimulus
Post
Time (minutes)

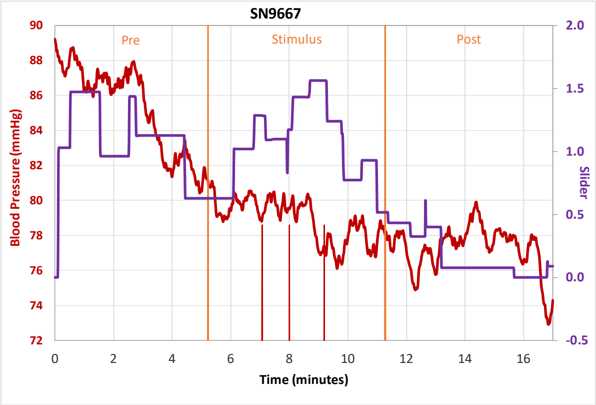
SN9667
Blood Pressure (mmHg)
Slider
Pre
Stimulus
Post
Time (minutes)

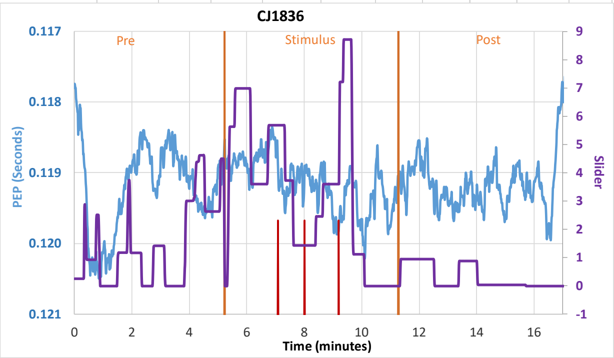
CJ1836
Pre
Stimulus
Post
PEP (Seconds)
Slider
Time (minutes)

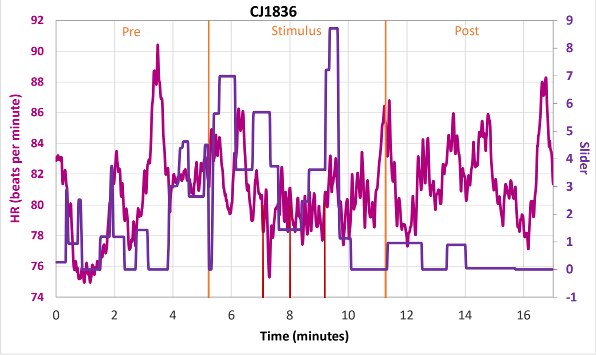
CJ1836
Pre
Stimulus
Post
HR (beats per minute)
Slider
Time (minutes)

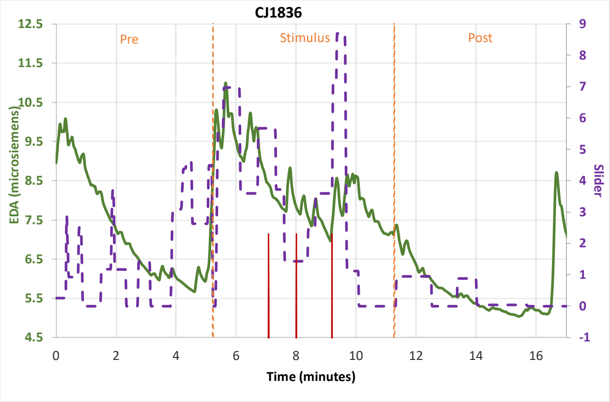
CJ1836
Pre
Stimulus
Post
EDA (microsiemens)
Slider
Time (minutes)

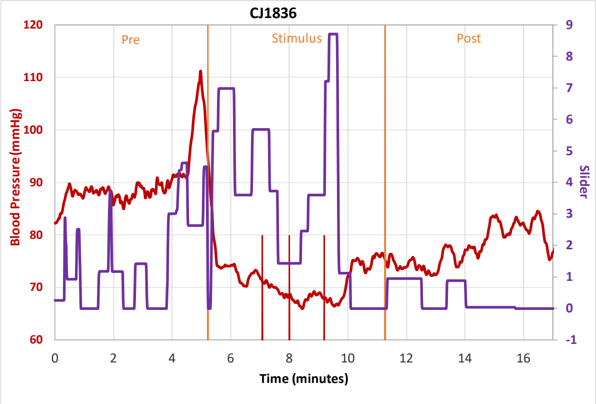
CJ1836
Pre
Stimulus
Post
Blood Pressure (mmHg)
Slider
Time (minutes)

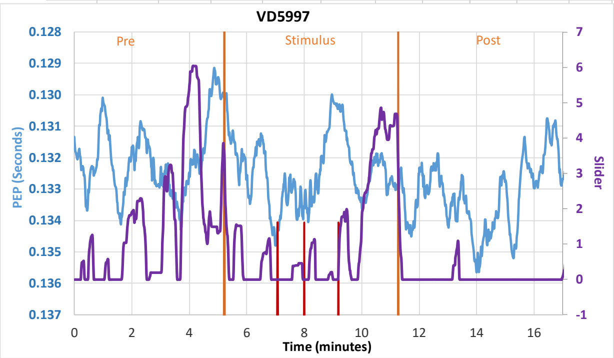
VD5997
Pre
Stimulus
Post
PEP (Seconds)
Slider
Time (minutes)

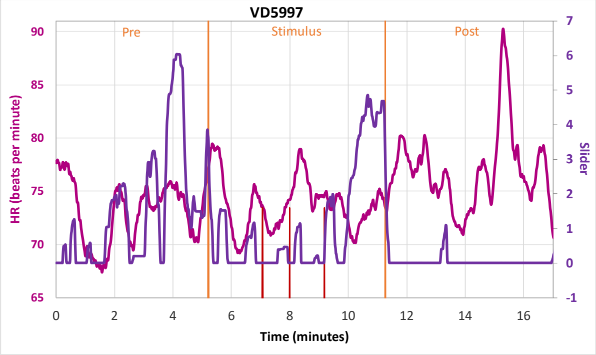
VD5997
Pre
Stimulus
Post
HR (beats per minute)
Slider
Time (minutes)

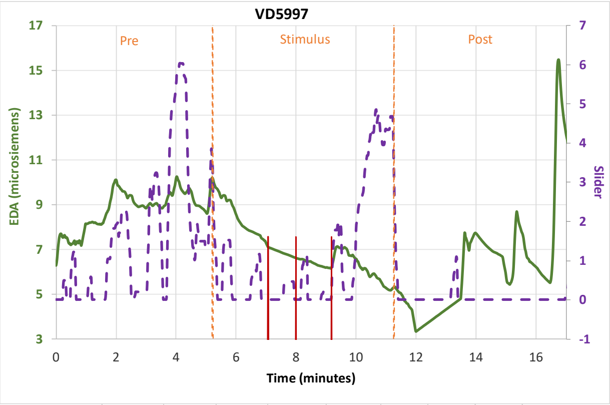
VD5997
Pre
Stimulus
Post
EDA (microsiemens)
Slider
Time (minutes)

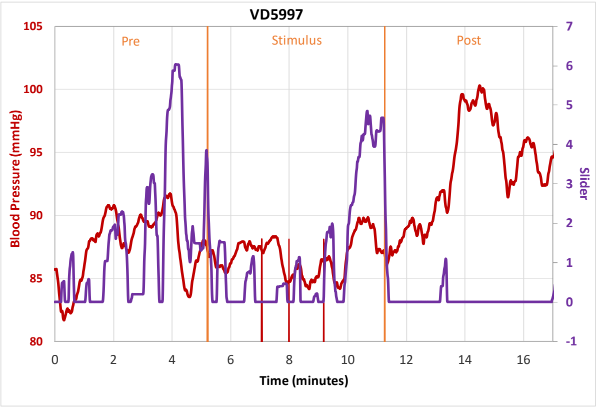
VD5997
Pre
Stimulus
Post
Blood Pressure (mmHg)
Slider
Time (minutes)

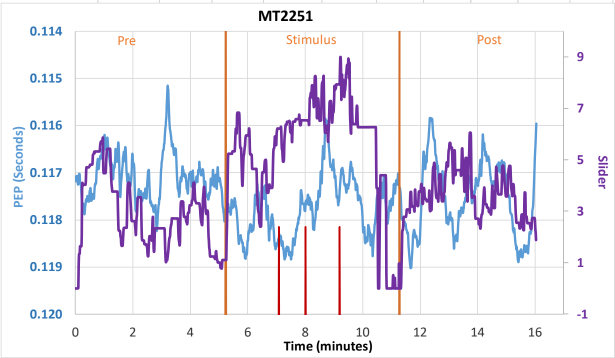
MT2251
Pre
Stimulus
Post
PEP (Seconds)
0.114
0.115
0.116
0.117
0.118
0.119
0.120
Slider
9
7
5
3
1
-1
0
2
4
6
8
10
12
14
16
Time (minutes)

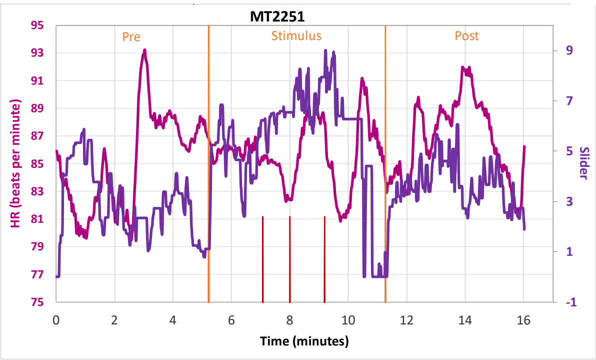
MT2251
Pre
Stimulus
Post
HR (beats per minute)
95
93
91
89
87
85
83
81
79
77
75
Slider
9
7
5
3
1
-1
0
2
4
6
8
10
12
14
16
Time (minutes)

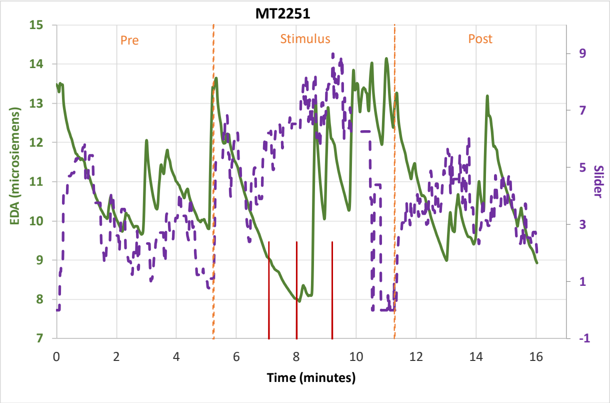
MT2251
Pre
Stimulus
Post
EDA (microsiemens)
15
14
13
12
11
10
9
8
7
Slider
9
7
5
3
1
-1
0
2
4
6
8
10
12
14
16
Time (minutes)

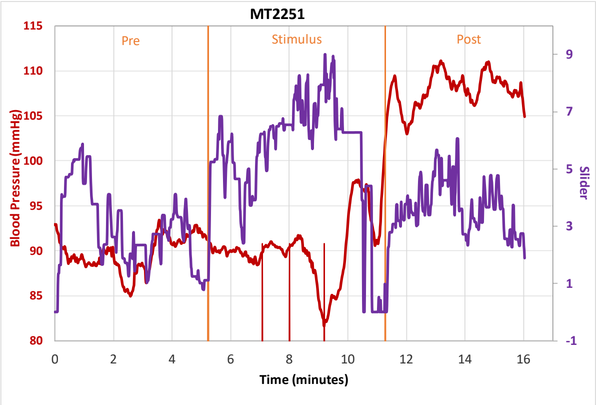
MT2251
Pre
Stimulus
Post
Blood Pressure (mmHg)
115
110
105
100
95
90
85
80
Slider
9
7
5
3
1
-1
0
2
4
6
8
10
12
14
16
Time (minutes)

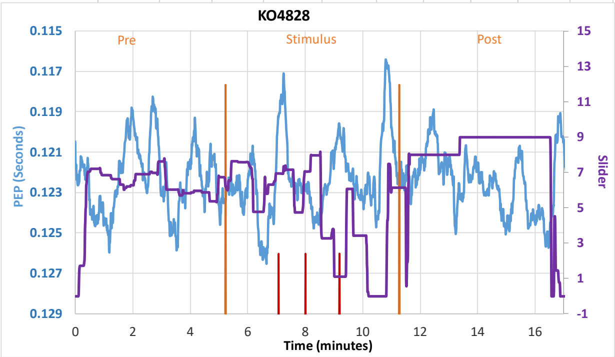
KO4828
Pre
Stimulus
Post
PEP (Seconds)
0.115
0.117
0.119
0.121
0.123
0.125
0.127
0.129
Slider
15
13
11
9
7
5
3
1
-1
0
2
4
6
8
10
12
14
16
Time (minutes)

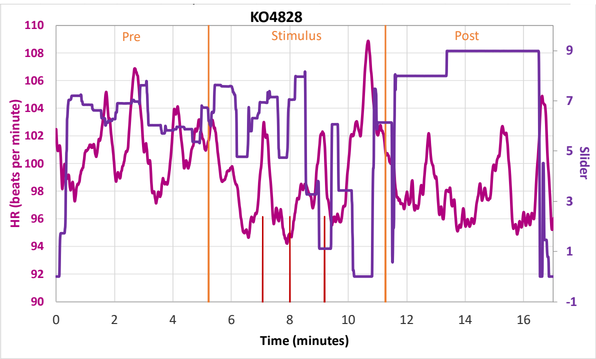
KO4828
Pre
Stimulus
Post
HR (beats per minute)
110
108
106
104
102
100
98
96
94
92
90
Slider
9
7
5
3
1
-1
0
2
4
6
8
10
12
14
16
Time (minutes)

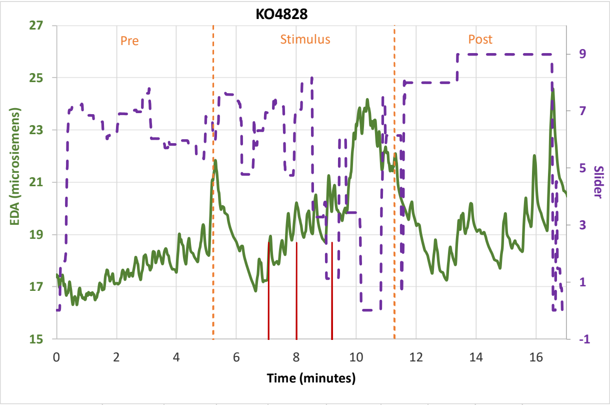
KO4828
Pre
Stimulus
Post
EDA (microsiemens)
27
25
23
21
19
17
15
Slider
9
7
5
3
1
-1
0
2
4
6
8
10
12
14
16
Time (minutes)

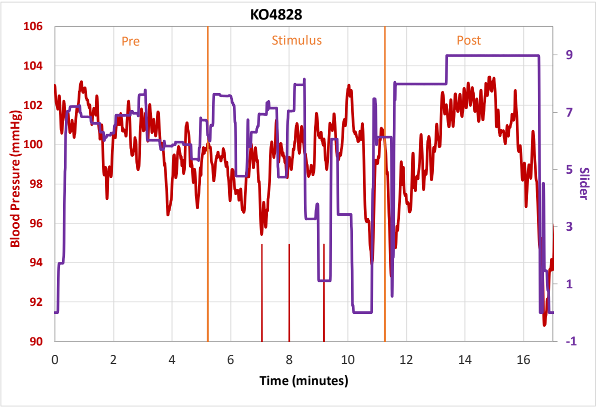
KO4828
Pre
Stimulus
Post
Blood Pressure (mmHg)
106
104
102
100
98
96
94
92
90
Slider
9
7
5
3
1
-1
0
2
4
6
8
10
12
14
16
Time (minutes)

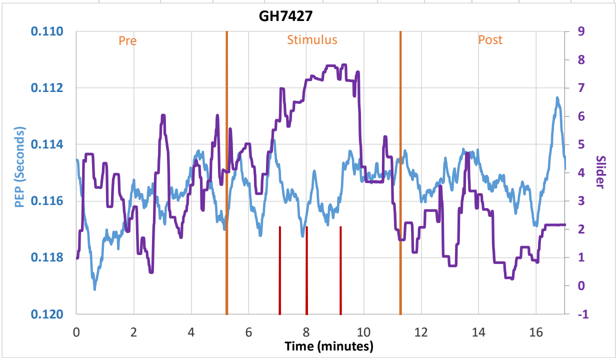
GH7427
Pre
Stimulus
Post
PEP (Seconds)
Slider
Time (minutes)

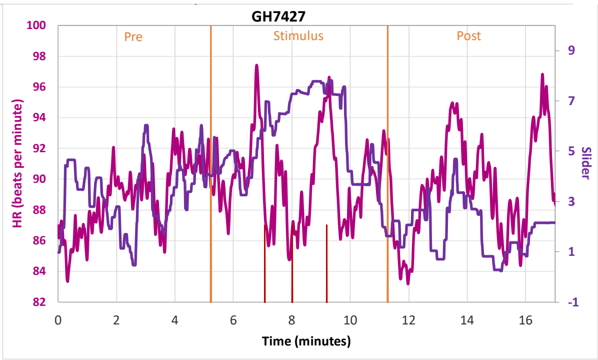
GH7427
Pre
Stimulus
Post
HR (beats per minute)
Slider
Time (minutes)

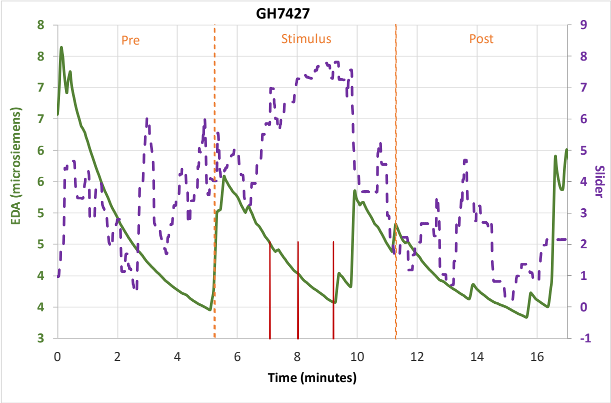
GH7427
Pre
Stimulus
Post
EDA (microsiemens)
Slider
Time (minutes)

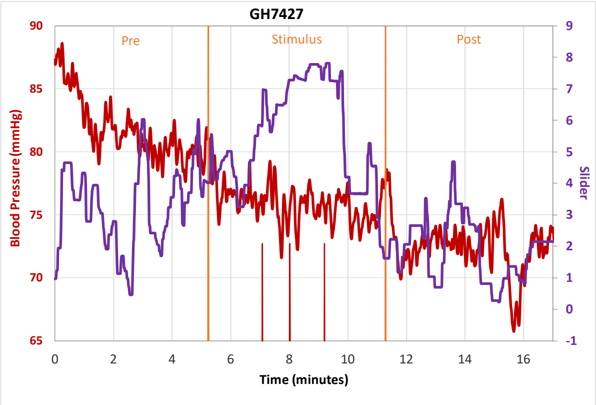
GH7427
Pre
Stimulus
Post
Blood Pressure (mmHg)
Slider
Time (minutes)

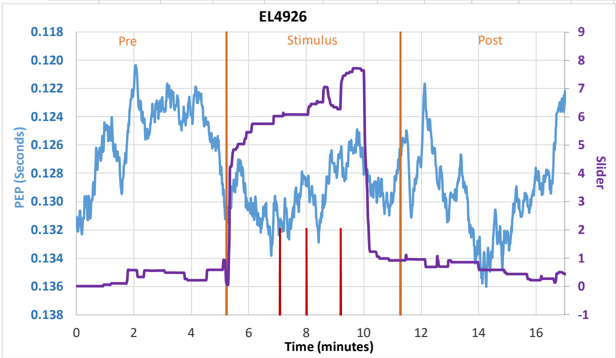
EL4926
Pre
Stimulus
Post
PEP (Seconds)
Slider
Time (minutes)

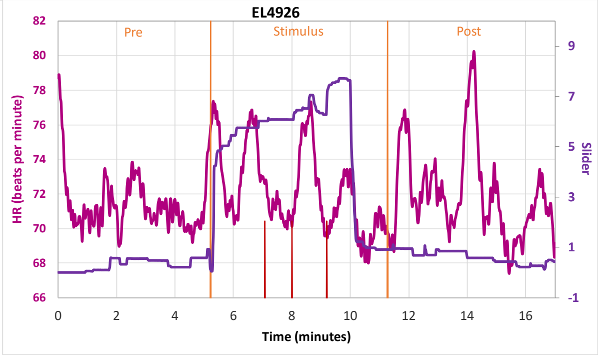
EL4926
Pre
Stimulus
Post
HR (beats per minute)
Slider
Time (minutes)

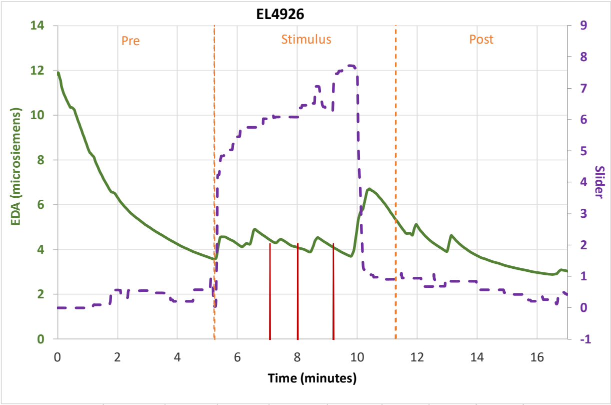
EL4926
Pre
Stimulus
Post
EDA (microsiemens)
Slider
Time (minutes)

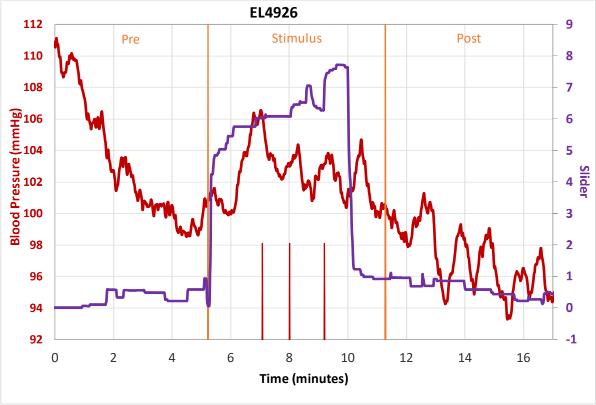
EL4926
Pre
Stimulus
Post
Blood Pressure (mmHg)
Slider
Time (minutes)

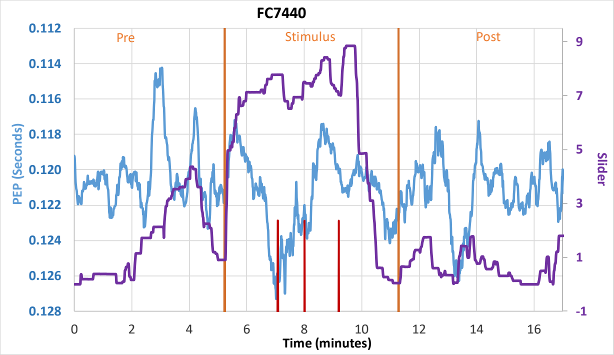
FC7440
Pre
Stimulus
Post
PEP (Seconds)
0.112
0.114
0.116
0.118
0.120
0.122
0.124
0.126
0.128
Slider
9
7
5
3
1
-1
0
2
4
6
8
10
12
14
16
Time (minutes)

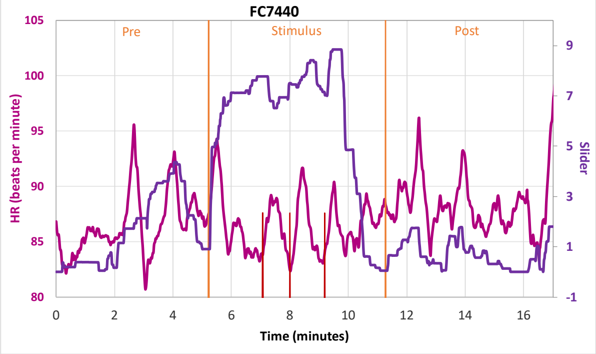
FC7440
Pre
Stimulus
Post
HR (beats per minute)
105
100
95
90
85
80
Slider
9
7
5
3
1
-1
0
2
4
6
8
10
12
14
16
Time (minutes)

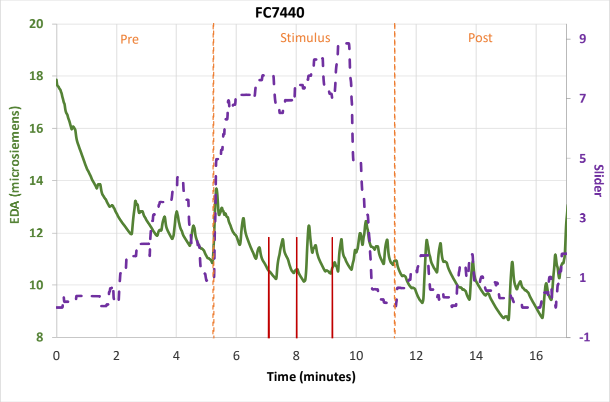
FC7440
Pre
Stimulus
Post
EDA (microsiemens)
20
18
16
14
12
10
8
Slider
9
7
5
3
1
-1
0
2
4
6
8
10
12
14
16
Time (minutes)

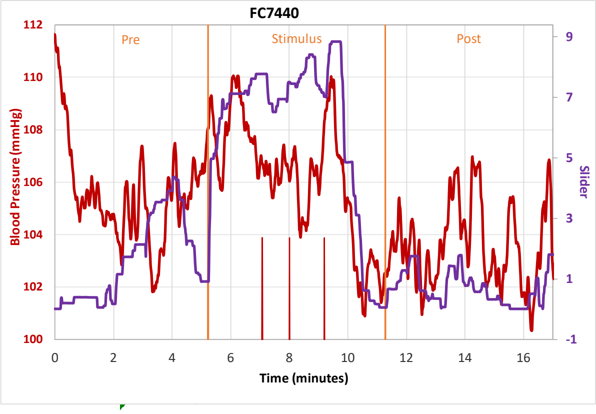
FC7440
Pre
Stimulus
Post
Blood Pressure (mmHg)
112
110
108
106
104
102
100
Slider
9
7
5
3
1
-1
0
2
4
6
8
10
12
14
16
Time (minutes)

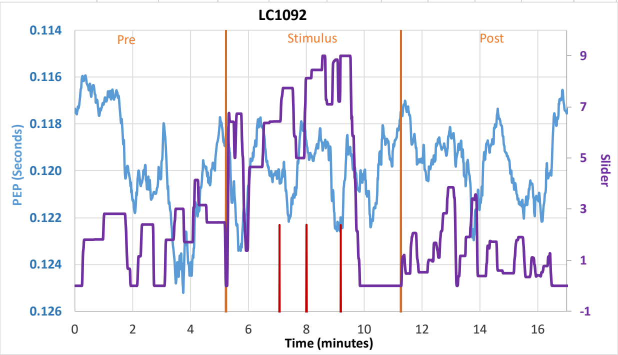
LC1092
Pre
Stimulus
Post
PEP (Seconds)
0.114
0.116
0.118
0.120
0.122
0.124
0.126
Slider
9
7
5
3
1
-1
0
2
4
6
8
10
12
14
16
Time (minutes)

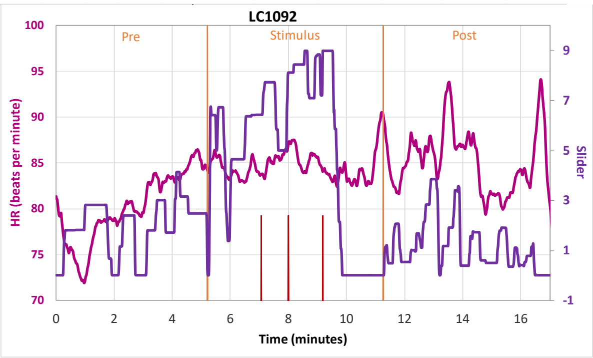
LC1092
Pre
Stimulus
Post
HR (beats per minute)
100
95
90
85
80
75
70
Slider
9
7
5
3
1
-1
0
2
4
6
8
10
12
14
16
Time (minutes)

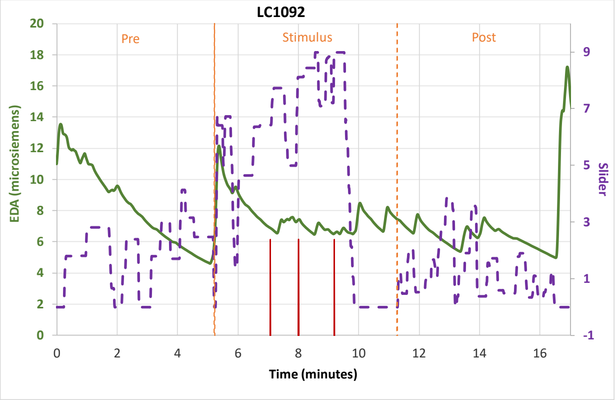
LC1092
Pre
Stimulus
Post
EDA (microsiemens)
20
18
16
14
12
10
8
6
4
2
0
Slider
9
7
5
3
1
-1
0
2
4
6
8
10
12
14
16
Time (minutes)

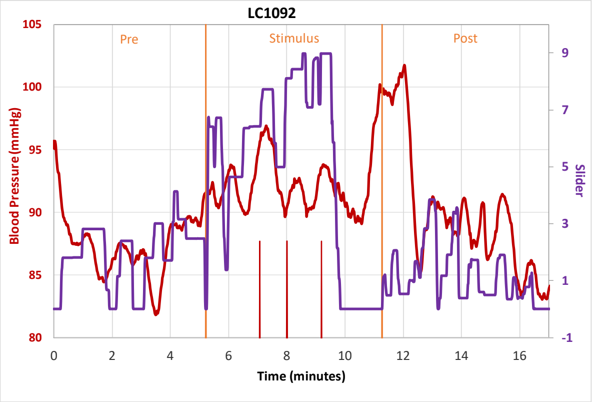
LC1092
Pre
Stimulus
Post
Blood Pressure (mmHg)
105
100
95
90
85
80
Slider
9
7
5
3
1
-1
0
2
4
6
8
10
12
14
16
Time (minutes)

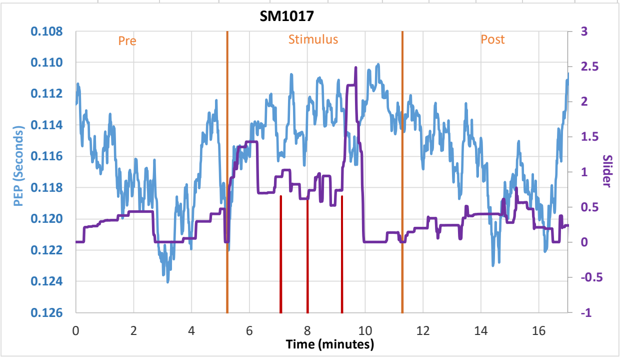
SM1017
Pre
Stimulus
Post
PEP (Seconds)
0.108
0.110
0.112
0.114
0.116
0.118
0.120
0.122
0.124
0.126
Slider
3
2.5
2
1.5
1
0.5
0
-0.5
-1
0 2 4 6 8 10 12 14 16
Time (minutes)

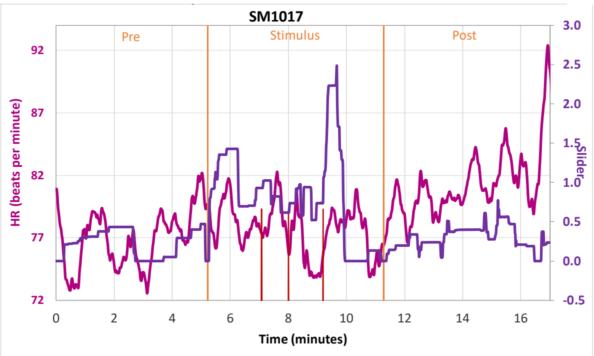
SM1017
Pre
Stimulus
Post
HR (beats per minute)
92
87
82
77
72
Slider
3.0
2.5
2.0
1.5
1.0
0.5
0.0
-0.5
0 2 4 6 8 10 12 14 16
Time (minutes)

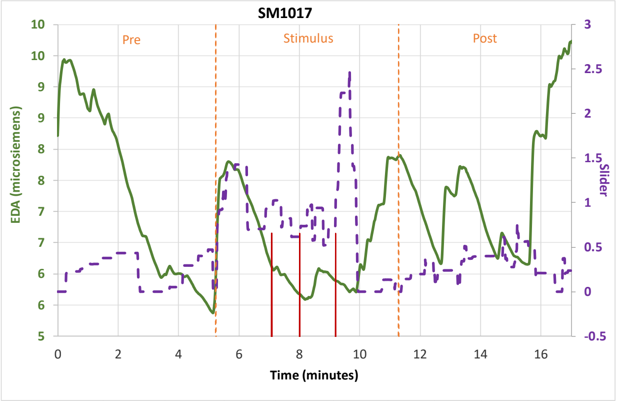
SM1017
Pre
Stimulus
Post
EDA (microsiemens)
10
10
9
9
8
8
7
7
6
6
5
Slider
3
2.5
2
1.5
1
0.5
0
-0.5
0 2 4 6 8 10 12 14 16
Time (minutes)

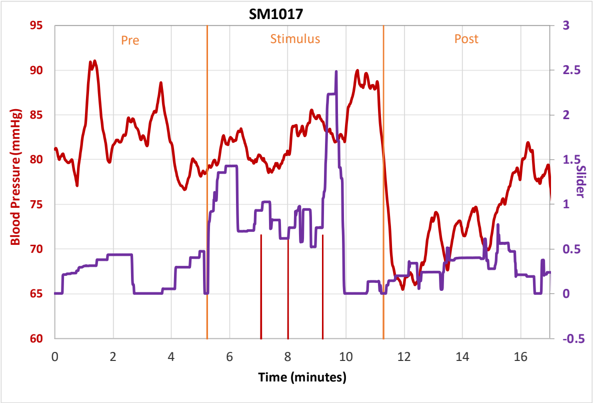
SM1017
Pre
Stimulus
Post
Blood Pressure (mmHg)
95
90
85
80
75
70
65
60
Slider
3
2.5
2
1.5
1
0.5
0
-0.5
0 2 4 6 8 10 12 14 16
Time (minutes)

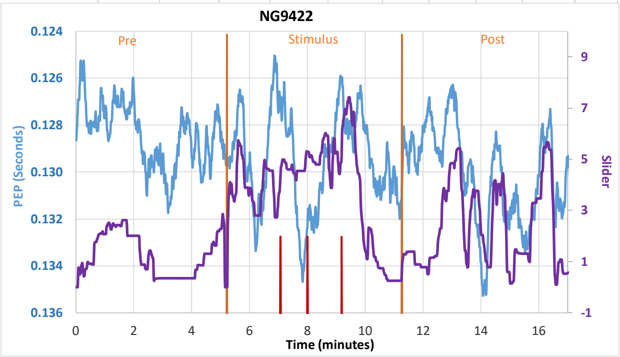
NG9422
Pre
Stimulus
Post
PEP (Seconds)
0.124
0.126
0.128
0.130
0.132
0.134
0.136
Slider
9
7
5
3
1
-1
0 2 4 6 8 10 12 14 16
Time (minutes)

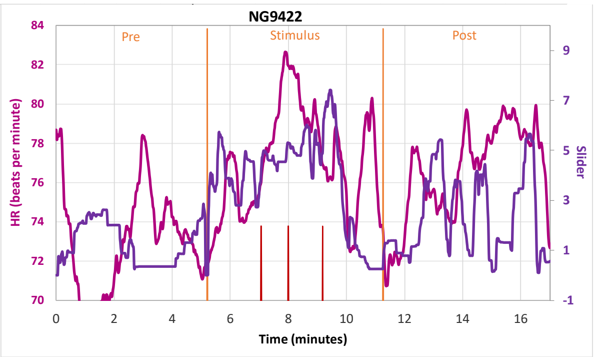
NG9422
Pre
Stimulus
Post
HR (beats per minute)
84
82
80
78
76
74
72
70
Slider
9
7
5
3
1
-1
0 2 4 6 8 10 12 14 16
Time (minutes)

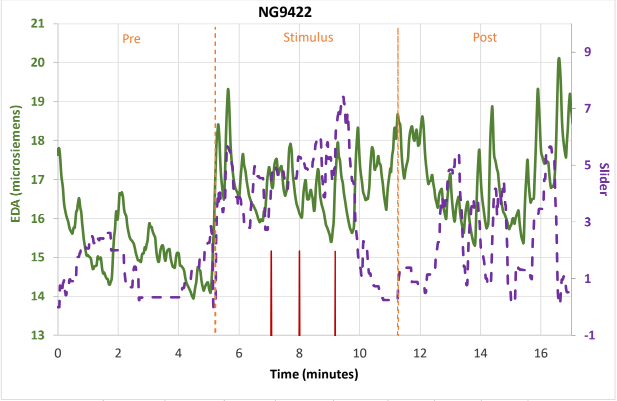
NG9422
Pre
Stimulus
Post
EDA (microsiemens)
21
20
19
18
17
16
15
14
13
Slider
9
7
5
3
1
-1
0 2 4 6 8 10 12 14 16
Time (minutes)

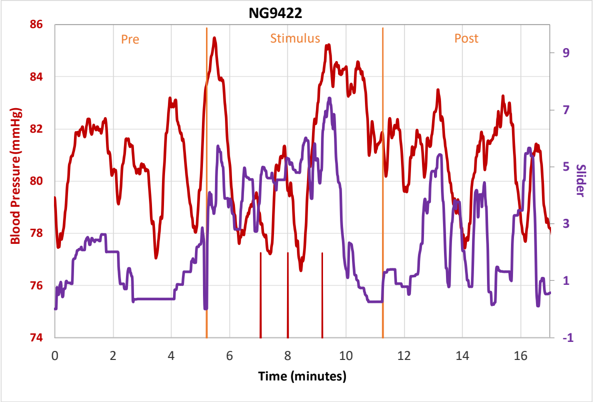
NG9422
Pre
Stimulus
Post
Blood Pressure (mmHg)
86
84
82
80
78
76
74
Slider
9
7
5
3
1
-1
0 2 4 6 8 10 12 14 16
Time (minutes)

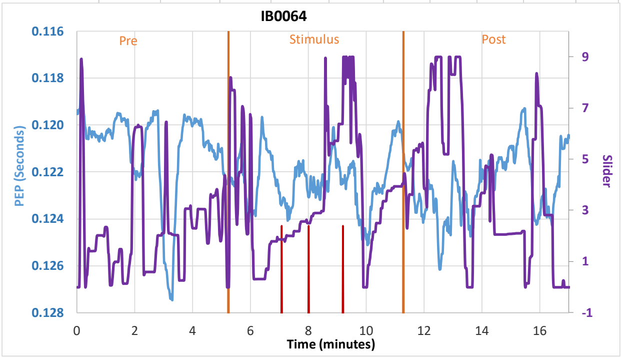
IB0064
Pre
Stimulus
Post
PEP (Seconds)
Slider
Time (minutes)

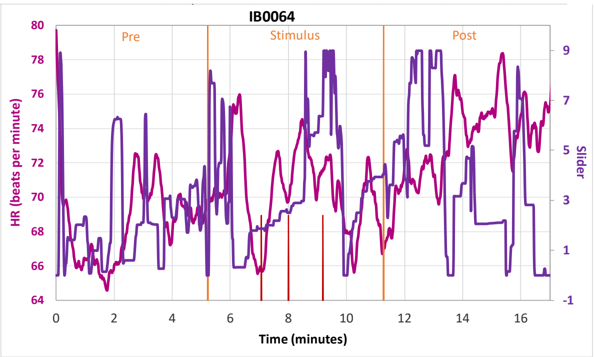
IB0064
Pre
Stimulus
Post
HR (beats per minute)
Slider
Time (minutes)

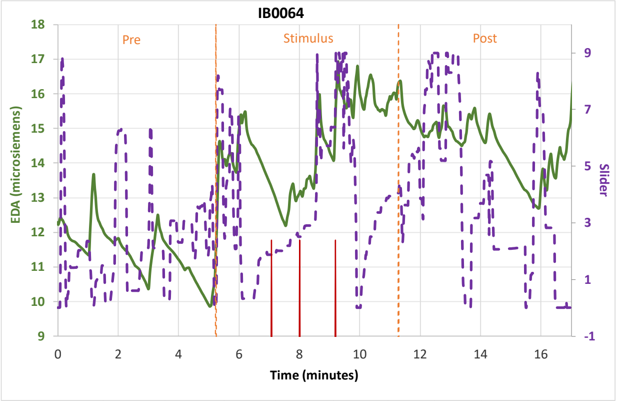
IB0064
Pre
Stimulus
Post
EDA (microsiemens)
Slider
Time (minutes)

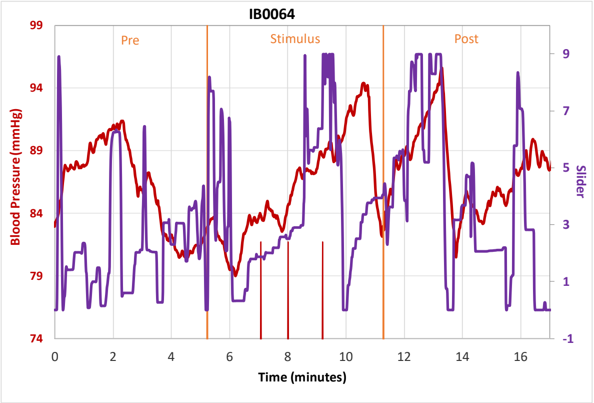
IB0064
Pre
Stimulus
Post
Blood Pressure (mmHg)
Slider
Time (minutes)

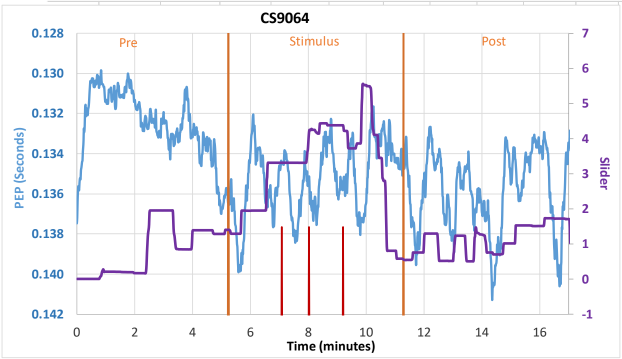
CS9064
Pre
Stimulus
Post
PEP (Seconds)
Slider
Time (minutes)

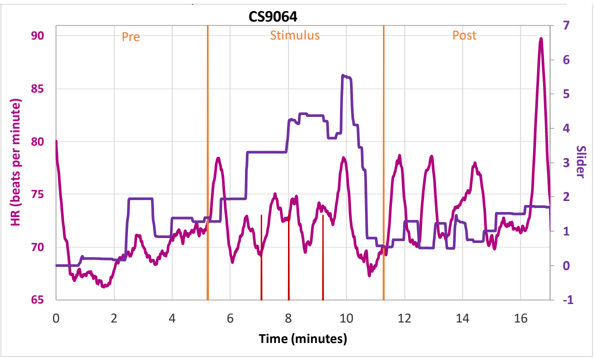
CS9064
Pre
Stimulus
Post
HR (beats per minute)
Slider
Time (minutes)

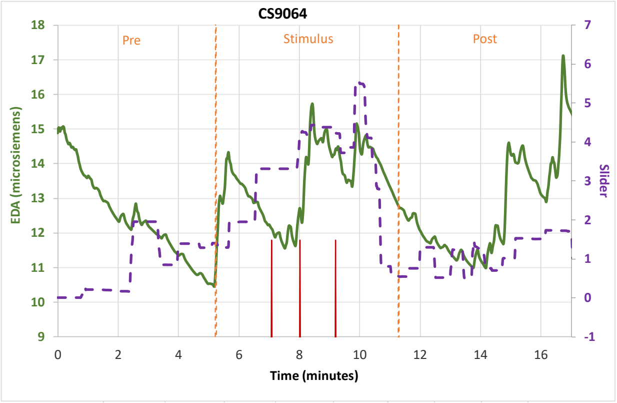
CS9064
Pre
Stimulus
Post
EDA (microsiemens)
Slider
Time (minutes)

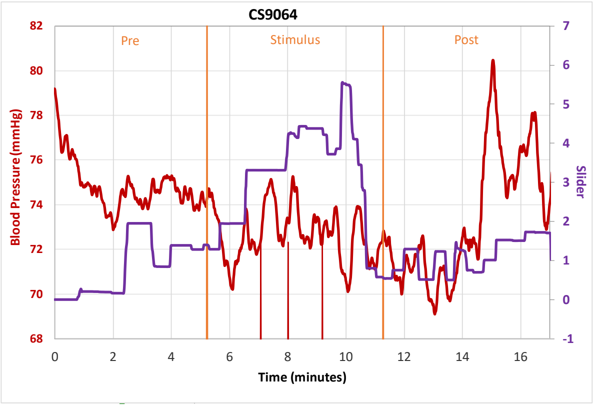
CS9064
Pre
Stimulus
Post
Blood Pressure (mmHg)
Slider
Time (minutes)

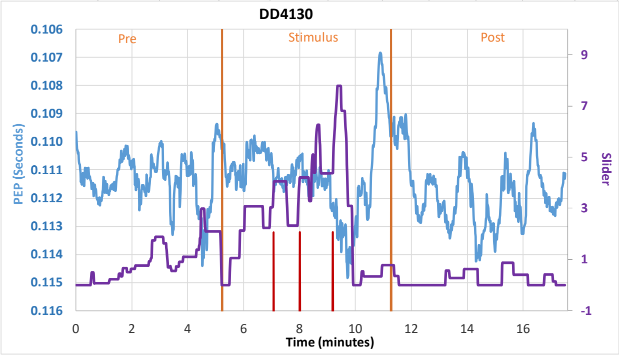
DD4130
Pre
Stimulus
Post
PEP (Seconds)
0.106
0.107
0.108
0.109
0.110
0.111
0.112
0.113
0.114
0.115
0.116
Slider
9
7
5
3
1
-1
0
2
4
6
8
10
12
14
16
Time (minutes)

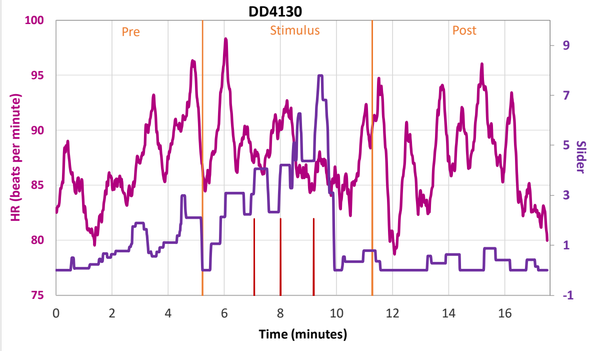
DD4130
Pre
Stimulus
Post
HR (beats per minute)
100
95
90
85
80
75
Slider
9
7
5
3
1
-1
0
2
4
6
8
10
12
14
16
Time (minutes)

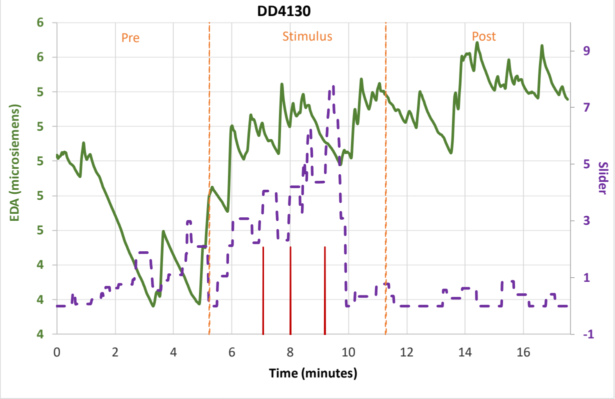
DD4130
Pre
Stimulus
Post
EDA (microsiemens)
6
6
5
5
5
5
5
4
4
4
Slider
9
7
5
3
1
-1
0
2
4
6
8
10
12
14
16
Time (minutes)

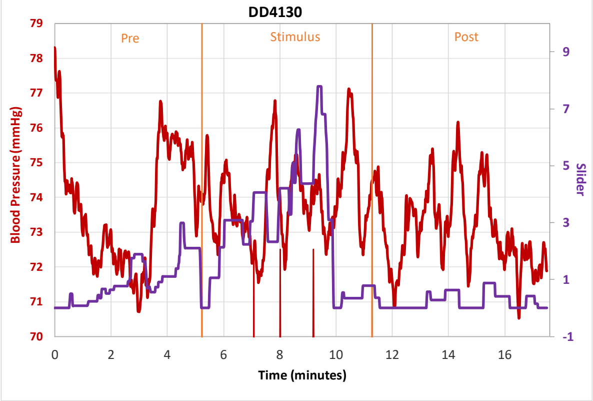
DD4130
Pre
Stimulus
Post
Blood Pressure (mmHg)
79
78
77
76
75
74
73
72
71
70
Slider
9
7
5
3
1
-1
0
2
4
6
8
10
12
14
16
Time (minutes)

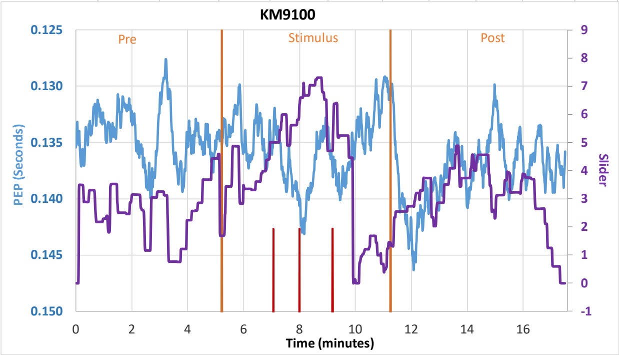
KM9100
Pre
Stimulus
Post
PEP (Seconds)
0.125
0.130
0.135
0.140
0.145
0.150
Slider
9
8
7
6
5
4
3
2
1
0
-1
0
2
4
6
8
10
12
14
16
Time (minutes)

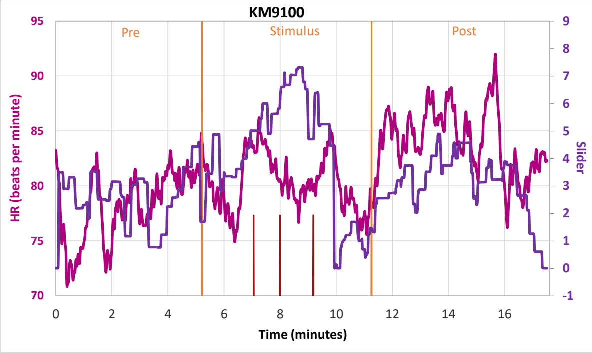
KM9100
Pre
Stimulus
Post
HR (beats per minute)
95
90
85
80
75
70
Slider
9
8
7
6
5
4
3
2
1
0
-1
0
2
4
6
8
10
12
14
16
Time (minutes)

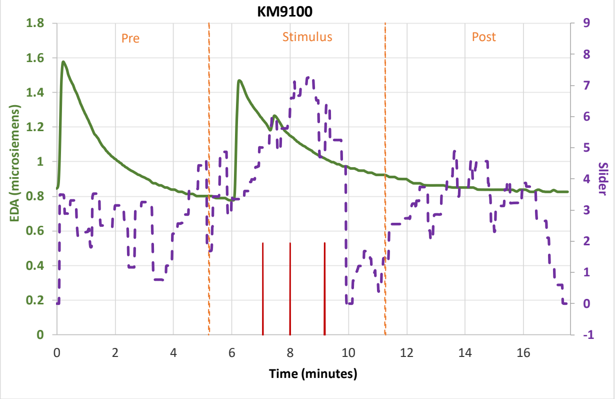
KM9100
Pre
Stimulus
Post
EDA (microsiemens)
1.8
1.6
1.4
1.2
1
0.8
0.6
0.4
0.2
0
Slider
9
8
7
6
5
4
3
2
1
0
-1
0
2
4
6
8
10
12
14
16
Time (minutes)

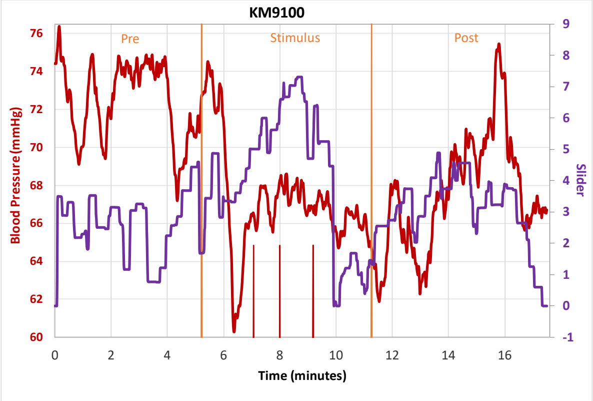
KM9100
Pre
Stimulus
Post
Blood Pressure (mmHg)
76
74
72
70
68
66
64
62
60
Slider
9
8
7
6
5
4
3
2
1
0
-1
0
2
4
6
8
10
12
14
16
Time (minutes)

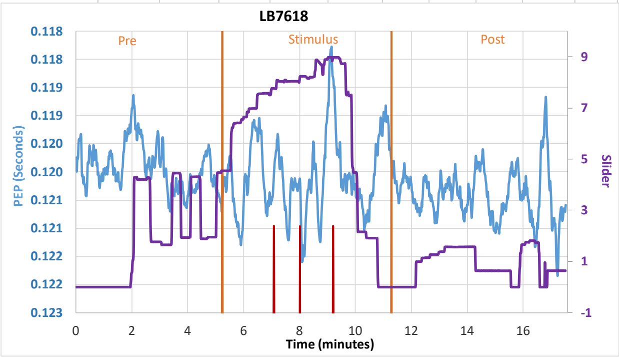
LB7618
Pre
Stimulus
Post
PEP (Seconds)
Slider
Time (minutes)

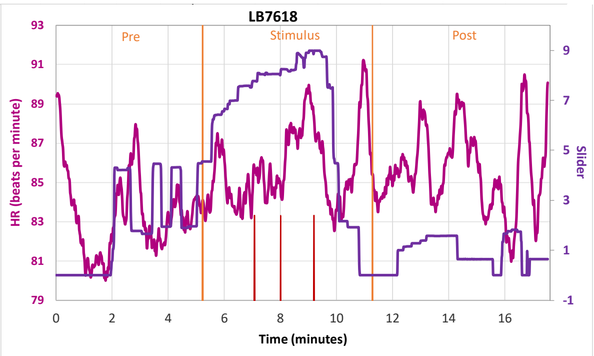
LB7618
Pre
Stimulus
Post
HR (beats per minute)
Slider
Time (minutes)

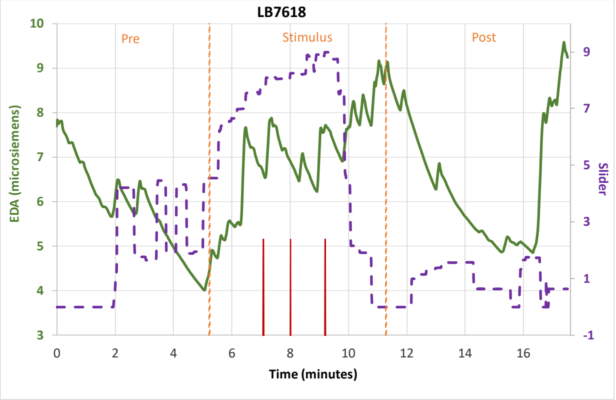
LB7618
Pre
Stimulus
Post
EDA (microsiemens)
Slider
Time (minutes)

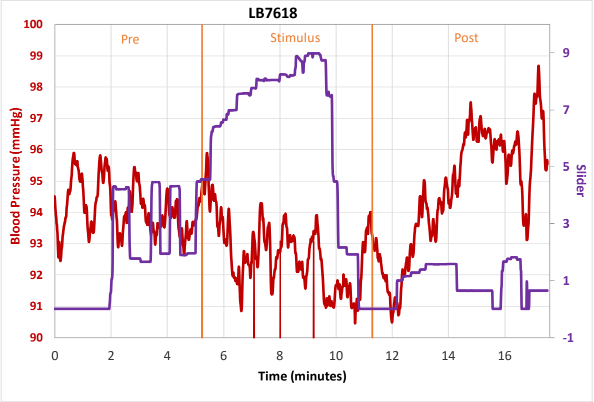
LB7618
Pre
Stimulus
Post
Blood Pressure (mmHg)
Slider
Time (minutes)

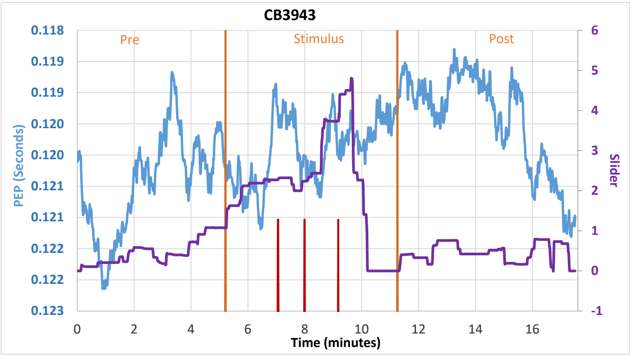
CB3943
Pre
Stimulus
Post
PEP (Seconds)
Slider
Time (minutes)

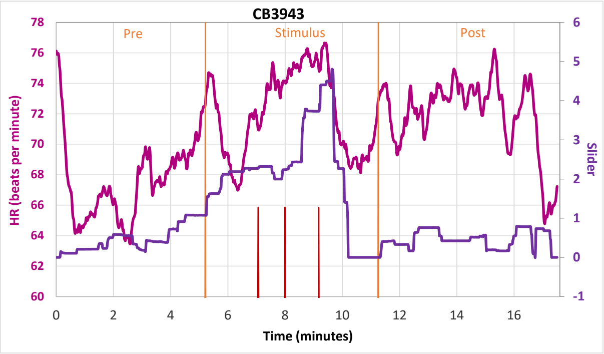
CB3943
Pre
Stimulus
Post
HR (beats per minute)
Slider
Time (minutes)

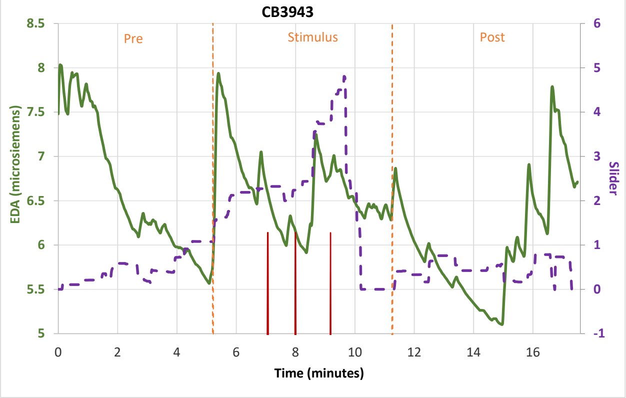
CB3943
Pre
Stimulus
Post
EDA (microsiemens)
Slider
Time (minutes)

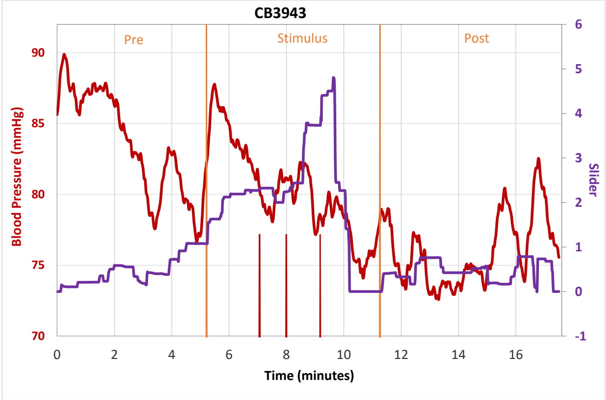
CB3943
Pre
Stimulus
Post
Blood Pressure (mmHg)
Slider
Time (minutes)

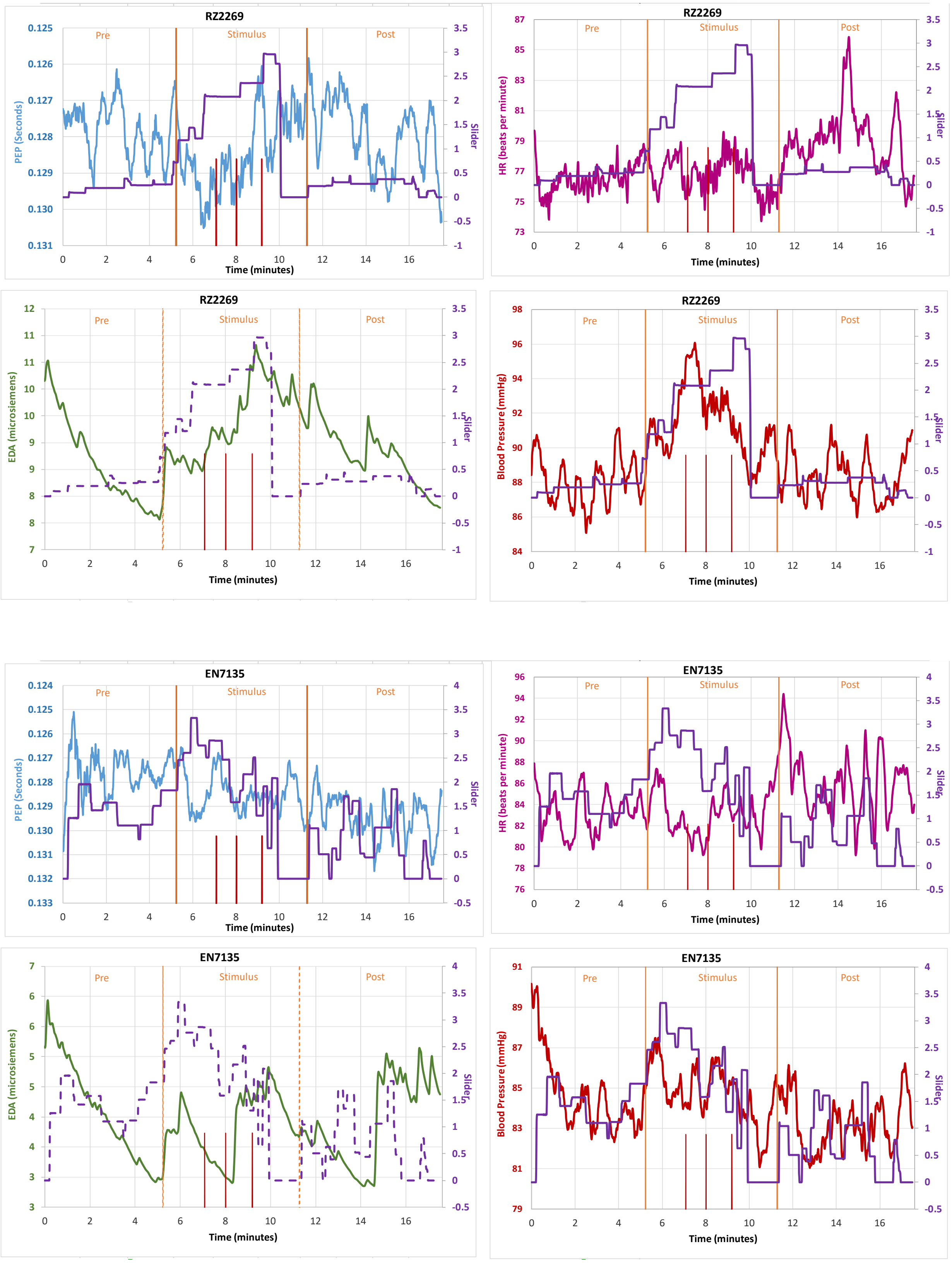
RZ2269
Pre
Stimulus
Post
PEP (Seconds)
Slider
Time (minutes)
HR (beats per minute)
EDA (microsiemens)
Blood Pressure (mmHg)
EN7135

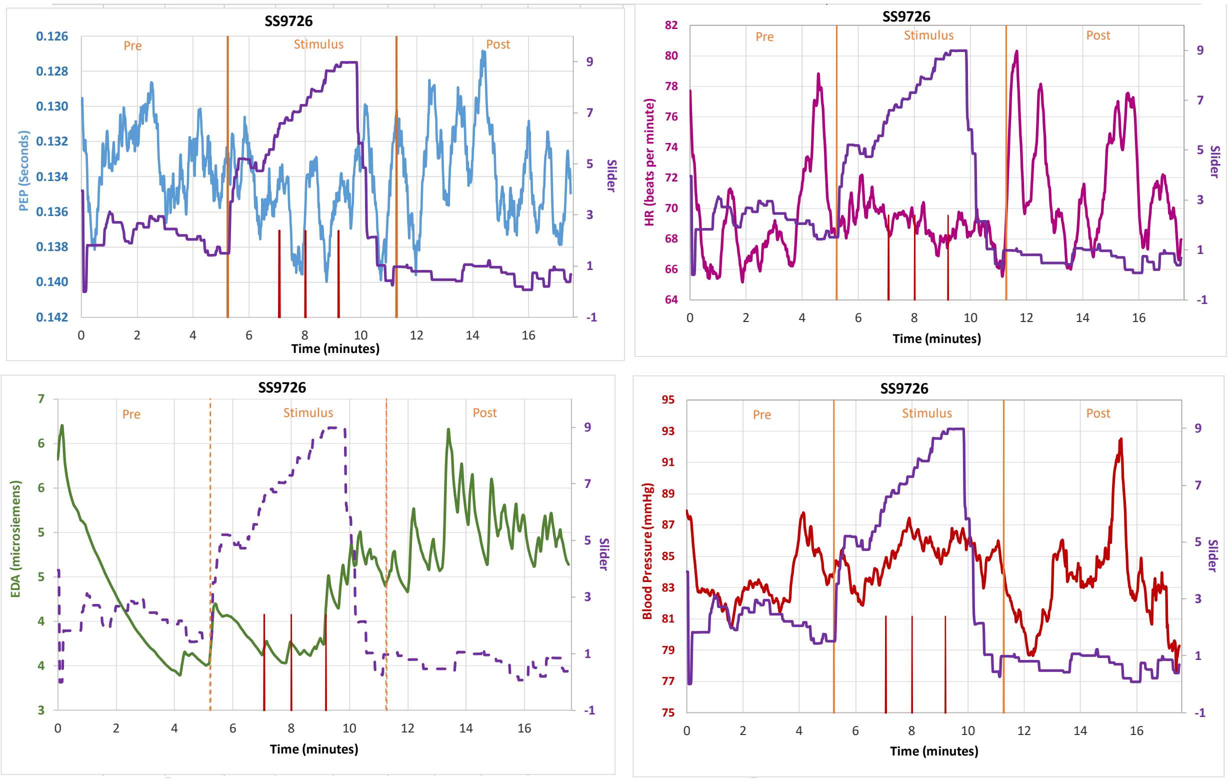
SS9726
Pre
Stimulus
Post
PEP (Seconds)
Slider
Time (minutes)
HR (beats per minute)
EDA (microsiemens)
Blood Pressure (mmHg)

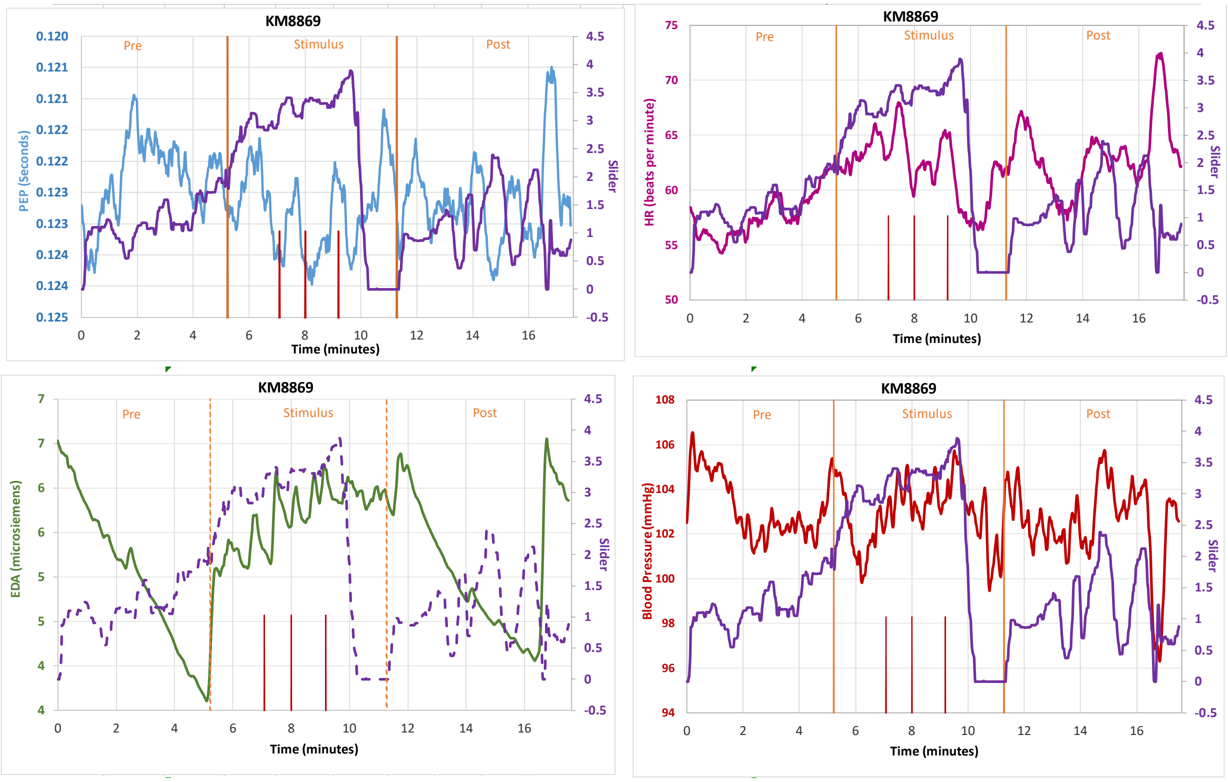
KM8869
Pre
Stimulus
Post
PEP (Seconds)
Slider
Time (minutes)
HR (beats per minute)
EDA (microsiemens)
Blood Pressure (mmHg)

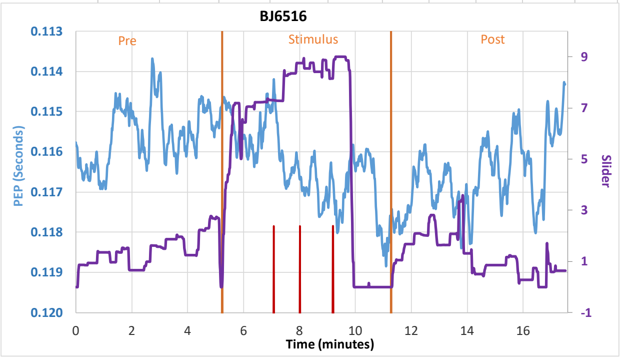
BJ6516
Pre
Stimulus
Post
PEP (Seconds)
Slider
Time (minutes)

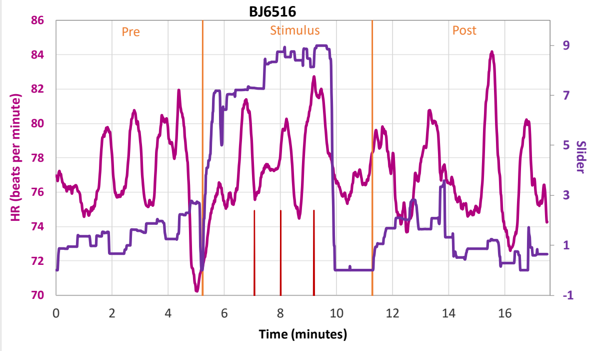
BJ6516
Pre
Stimulus
Post
HR (beats per minute)
Slider
Time (minutes)

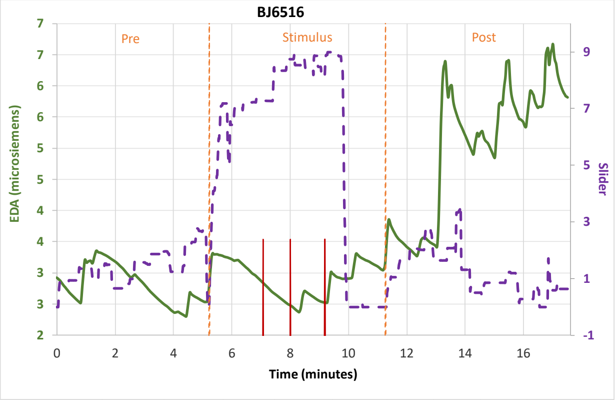
BJ6516
Pre
Stimulus
Post
EDA (microsiemens)
Slider
Time (minutes)

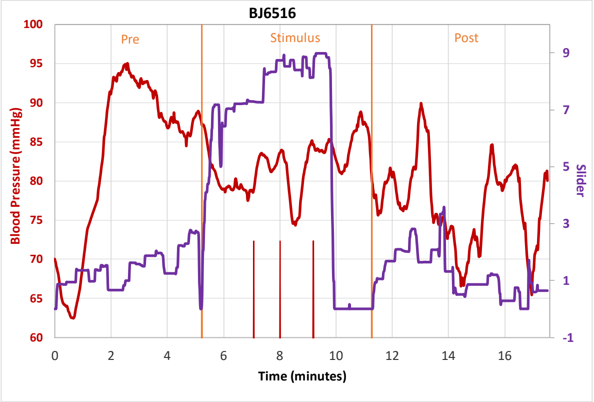
BJ6516
Pre
Stimulus
Post
Blood Pressure (mmHg)
Slider
Time (minutes)

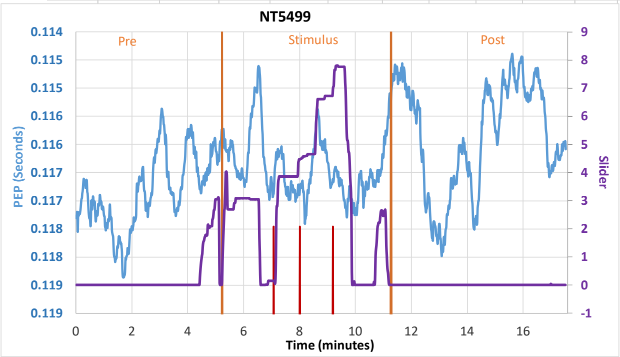
NT5499
Pre
Stimulus
Post
PEP (Seconds)
Slider
Time (minutes)

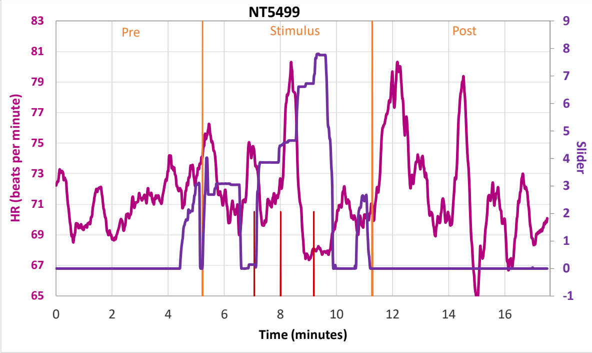
NT5499
Pre
Stimulus
Post
HR (beats per minute)
Slider
Time (minutes)

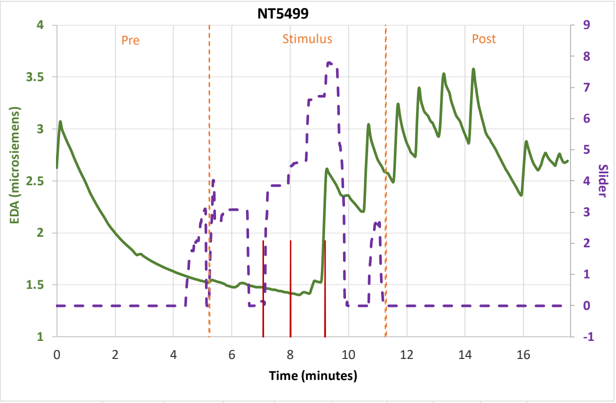
NT5499
Pre
Stimulus
Post
EDA (microsiemens)
Slider
Time (minutes)

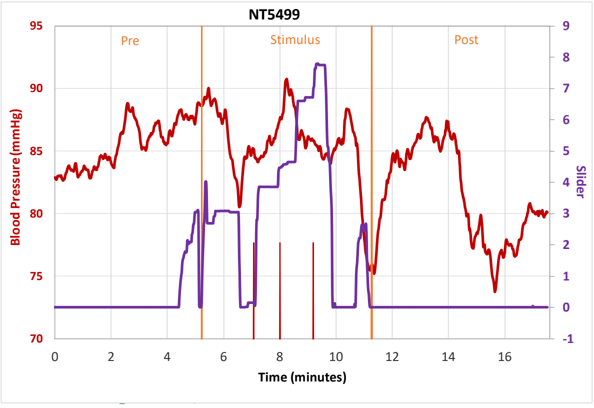
NT5499
Pre
Stimulus
Post
Blood Pressure (mmHg)
Slider
Time (minutes)

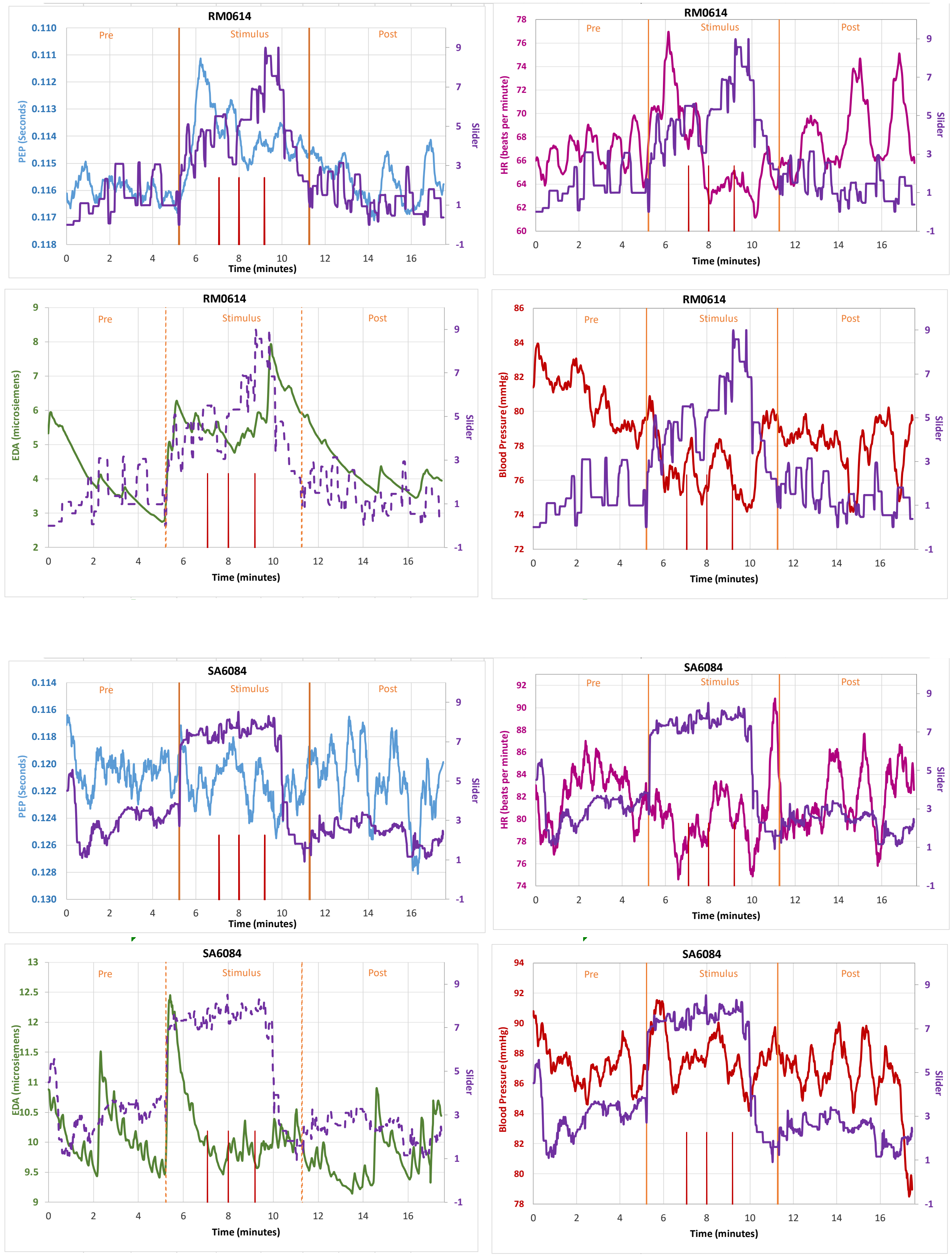
RM0614
Pre
Stimulus
Post
PEP (Seconds)
Slider
Time (minutes)
HR (beats per minute)
EDA (microsiemens)
Blood Pressure (mmHg)
SA6084
